# NbIT - A New Information Theory-Based Analysis of Allosteric Mechanisms Reveals Residues that Underlie Function in the Leucine Transporter LeuT

**DOI:** 10.1371/journal.pcbi.1003603

**Published:** 2014-05-01

**Authors:** Michael V. LeVine, Harel Weinstein

**Affiliations:** 1 Department of Physiology and Biophysics, Weill Cornell Medical College of Cornell University (WCMC), New York, New York, United States of America; 2 HRH Prince Alwaleed Bin Talal Bin Abdulaziz Alsaud Institute of Computational Biomedicine, Weill Cornell Medical College of Cornell University, New York, New York, United States of America; Icahn School of Medicine at Mount Sinai, United States of America

## Abstract

Complex networks of interacting residues and microdomains in the structures of biomolecular systems underlie the reliable propagation of information from an input signal, such as the concentration of a ligand, to sites that generate the appropriate output signal, such as enzymatic activity. This information transduction often carries the signal across relatively large distances at the molecular scale in a form of allostery that is essential for the physiological functions performed by biomolecules. While allosteric behaviors have been documented from experiments and computation, the mechanism of this form of allostery proved difficult to identify at the molecular level. Here, we introduce a novel analysis framework, called N-body Information Theory (NbIT) analysis, which is based on information theory and uses measures of configurational entropy in a biomolecular system to identify microdomains and individual residues that act as (i)-channels for long-distance information sharing between functional sites, and (ii)-coordinators that organize dynamics within functional sites. Application of the new method to molecular dynamics (MD) trajectories of the occluded state of the bacterial leucine transporter LeuT identifies a channel of allosteric coupling between the functionally important intracellular gate and the substrate binding sites known to modulate it. NbIT analysis is shown also to differentiate residues involved primarily in stabilizing the functional sites, from those that contribute to allosteric couplings between sites. NbIT analysis of MD data thus reveals rigorous mechanistic elements of allostery underlying the dynamics of biomolecular systems.

## Author Summary

We developed the new information theory-based analysis framework presented here, NbIT analysis, for the study of allosteric mechanisms in biomolecular systems from Molecular Dynamics trajectories. The illustrative application of NbIT to the analysis of the occluded state in the bacterial transporter LeuT, produced a quantitative representation of the allosteric behavior, and identified intramolecular channels that enable the long-distance information transmission. Our findings, identifying the roles of specific residues in the communication of the allosteric information, were validated by the recognition of residues that have been previously shown to play functional roles in this very well studied system. In addition, we show that application of NbIT analysis leads to the discrimination of functional roles by differentiating between residues that are essential to the dynamics within functional sites (e.g., the substrate binding sites), and residues whose role is to communicate between such functional sites. These results demonstrate that the information theoretical analysis presented here is a powerful tool for quantifying complex allosteric behavior in biomolecular systems and for identifying the crucial components underlying those behaviors.

## Introduction

The propagation of information over long distances at the molecular and cellular scale is essential for the expedient and efficient regulation of cell function. For example, biomolecular systems involved in cell signaling can detect an input signal, such as the concentration of a ligand, ion, or biomolecule, and transmit that signal through molecular interaction networks to specialized sites such as ligand release sites in transporters, or catalytic sites in enzymes. The intramolecular propagation of information between distant parts of the biomolecules is now known as allostery, and was first discussed in the context of end-product inhibition by Monod, Changeux, and Jacob [1]. It is now well documented that such allosteric communication underlies function in a vast number of biomolecular systems, to the point that it is believed that nearly all proteins display some level of allosteric behavior [2].

**Figure 1:**
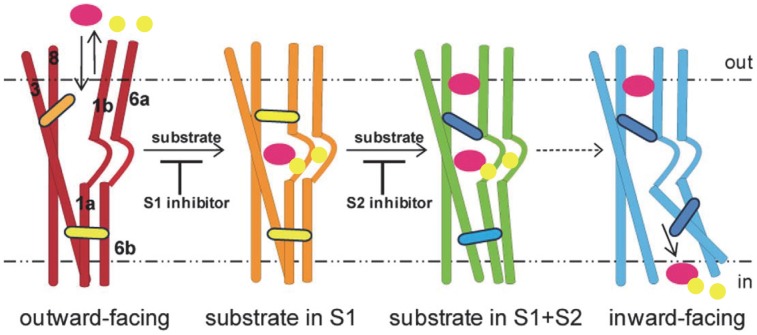
Representation of the states in the transport cycle of an NSS transporter. In this model, the transporter begins in an outward-open state (red), which can bind Na+ (yellow) and substrate (purple) in the primary site (S1) and then transition to a substrate-bound occluded state (orange). This state can bind in the S2 site, either inhibitors, such as TCAs, which block substrate release, or substrates (green), which produce release of Na+ and substrate from S1 (blue). Reproduced and modified with permission from [12].

The development of new experimental and computational techniques has recently made it possible to observe allosteric behavior with high resolution. The prototypical member of the family of neurotransmitter∶sodium symporters (NSS), the bacterial transporter LeuT analyzed here with the new approach, has been particularly well studied, and the results from many experimental and computational investigations suggest that transport is driven by a complex allosteric mechanism spanning the entire length of the transporter. The transport cycle is believed to adhere to the stages of the canonical alternating access model [3] involving transitions between at least three distinct conformational states: an *extracellular-open, outward-facing state* [4] in which the symported ions and substrate are bound, followed by an *occluded state* [5] that shields the transported substrate from the extracellular environment from which it came, and an *intracellular-open, inward-facing state* [4] which can then release the substrate. From single molecule FRET (smFRET) experiments carried out on LeuT, a number of transport-related structural transitions were identified in the intracellular gate region that occludes the substrate from the cytoplasm [6], and these were shown to be modulated by binding events at the extracellular end [7], [8]. Crystallographic studies have also revealed that a second binding site in the extracellular vestibule (termed S2) is the target of several transport inhibitors (including many of the psycho-active drugs acting on the cognate NSS neurotransmitter transporters) [9], [10], and biochemical and computational evidence suggests that the release of substrate is allosterically connected to the binding of a second substrate in this site [11]–[13]. These results bring to light the cross-talk between several allosterically coupled domains in the transport mechanism of NSS transporters, and suggest that modulation of these domains can both facilitate and hinder function. The schematics in Fig. 1 depict the transport cycle that takes into account the recently described allosteric roles of substrate in bound in the primary site, S1, and in S2. Still lacking, however, is a suitable quantitative formulation of the channels through which information can be communicated from one part of the molecule to another in the individual states of the transporter that constitute the transport cycle.

Indeed, the specific process of allosteric signal propagation in a molecular system through intramolecular interactions has not yet been subjected to experimental measurements, although the allosteric effect can be observed experimentally from the apparent relation between distal parts of a macromolecule. To date, there are no experimental methods capable of specifically and definitively defining the role of the intramolecular interactions involved in propagating allostery. Most proposed mechanisms are descriptions of series of local rearrangements presumed (but not demonstrated) to be causally sequential – a specific, quantitative definition of the information flow does not exist. For example, a successful experimental method for determining residues that are coupled to ligand binding, the mutant cycle analysis [14], while able to quantify thermodynamic coupling at a distance, still relies on these sequential descriptions to propose the underlying mechanism of propagation. For these reasons, theoretical and computational approaches to determine if and how distant domains are coupled within a single state have been proposed [15]–[17], with the intention of using atomic-level insight which in unavailable experimentally to propose physical mechanisms.

In developing the new analysis described herein, we reasoned that if the macro (i.e., whole molecule) states of two domains are coupled (e.g., if the population of an open and a closed state of the intracellular gate, as well as the transitions between them, are coupled to the occupancy state of the substrate sites), their micro (i.e., component) states would also exhibit coupling (e.g., the fluctuations within the closed state of the intracellular gate would be coupled to the fluctuations within the bound state of the substrate site). Because this needs to be demonstrated rigorously, we undertook the investigation of the information coupling between such molecular domains known to have functional significance in LeuT in a particular state. Investigating the mechanics of the protein in one such state of the transport cycle enables the identification of potential allosteric channels that may be used to propagate information in general.

In previous computational approaches to solve this problem, the focus is on modeling single states of a protein as an interaction network obtained by assigning nodes to residues and parameterizing edges using either crystal structure contacts [17]–[19], or pair-wise atomic fluctuation correlations from Molecular Dynamics [15], [16], [20], [21]. The advantage of such networks is that the parameterization of edges in the interaction network is computationally reasonable (only requiring structures and reasonable simulation time) and appropriate network theoretical approaches exist, mostly based on graph theory, to achieve the identification of (a)-paths through the network that may propagate allosteric effects [22], and (b)-community structures that may act as information hubs or subnetworks [15], [23]. However, analysis of allosteric mechanisms with these methods must be considered incomplete, because only pair-wise correlation is considered, and not the other N-body correlated motions. This is a drawback, because correlated motions at the N-body level are both present in, and required for, a complex collective behavior such as allostery (see illustrative example in “Supporting Discussion 1: Efficient Information Transmission” and Fig. S1 in File S1). The new method we describe here identifies communication channels within allosteric biomolecular systems through information theory-based analysis of N-body collective dynamics determined from the configurational entropy of the system.

We describe the new method, which we call **N**-**b**ody **I**nformation **T**heory (NbIT) analysis, through the application to a structurally defined state of LeuT, the occluded state (3GJD) described above [24], [25]. A mechanistic scheme for the substrate-modulated gating dynamics in such a LeuT state can be considered intuitively as an information theoretical communication process. In such a mechanistic scheme, the binding signal is detected by the substrate site(s), which then acts as a *transmitter* that sends the information through an intramolecular channel spanning the transmembrane region, to the *receiver*. In the case of LeuT, the receiver is the intracellular gate that needs to open in order for the transported substrate to be eventually released to the cytoplasm. Based on this representation in the frame of information transmission through the intramolecular channel, the goal of identifying the allosteric mechanism connecting the two distally positioned functional sites, translates into an analysis that can identify the specific residues that compose the intramolecular communication channel by identifying patterns of multi-body information sharing.

**Figure 2:**
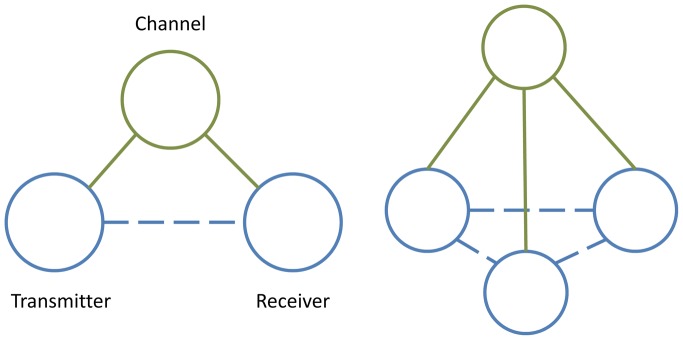
Allosteric information communication. Left: Illustration of the basic receiver-channel-transmitter system. The receiver and transmitter (blue) interact separately with the channel (green) via direct interaction (green lines). This leads to an indirect, allosteric interaction between the receiver and transmitter (blue dashed line). Right: several receiver-transmitter pairs are coupled by the same channel, which leads to coordination of the behavior of all the receivers/transmitters.

The new NbIT analysis method presented here utilizes a generalization of the concept of *co-information* (also known as interaction information) [26]–[29], an information theoretical measure which enables a description of the contribution that a variable makes to the *mutual information* shared between two other variables. We extend *co-information* to describe the contribution of a variable to the more general measure of *total correlation*, in order to describe the contribution of a variable to information shared between any number of other variables. The advantage of this extension beyond the *mutual information* [30], which describes the information shared by 2 variables, to the *total correlation* (also known as multi-information) [31]–[33], is that the latter describes the total amount of information shared between a set of N variables through all possible n-body correlations ranging from 2 to N. This generalization of *co-information* is called *coordination information*, and it can identify residues that coordinate the N-body correlated motions present within a set of residues, such as functional sites, by playing the role of channel across many different transmitter-receiver combinations (see Fig. 2, right). We show that the use of *coordination information* reveals how global motions within functional domains are modulated allosterically by distant sites. In addition, by developing another information theoretical measure, the *mutual coordination information*, we are able to identify channels that propagate coordination information. This is illustrated specifically when NbIT is applied to the analysis of configurational entropies estimated from Molecular Dynamics (MD) simulations of LeuT starting from the occluded state crystal structure. Thus, the molecular level mechanism of information transduction that emerges from the analysis describes how several already known allosteric couplings are generated. Specifically, we examine the communication within the ligand-bound occluded state in which the intracellular gate is closed. Importantly, we show that within this state, we can identify the specific contribution to the allosteric mechanism of “functional residues” (both previously known and newly revealed here). Moreover, we contrast the roles of such “functional residues” to those of other residues that contribute only to the stability of the functional sites, but not the allosteric coupling. The detailed illustration shows how NbIT analysis applied to a functionally distinct macrostate for which the configurational entropy can be estimated reveals the allosteric channels conducive to a key component of the functional mechanism. This example further suggests that when the same NbIT analysis is applied to an ensemble of states of a particular molecular system such as the LeuT, which can include several functionally distinct macrostates, the results should reveal the complement of allosteric channels conducive to the functional mechanism of that molecular system.

## Methods

### Trajectories from Molecular Dynamics Simulations

Two separate trajectories of the same LeuT structure were analyzed with the NbIT method. The LeuT_POPE/POPG_ trajectory is a simulation of the occluded LeuT structure [24] (PDB ID 3GJD) bound to the two sodium ions and leucine, but with the octyl-glucoside (OG) detergent molecule removed, which has been described previously [25]. The LeuT_MNG-3_ trajectory is for the same LeuT structure simulated in lauryl maltose-neopentyl glycol (MNG-3), a detergent known for its excellent stabilization of transmembrane proteins, including LeuT, in micellar environments [34], [35]. Both simulations were run at in an NPT ensemble at 310 K temperature using the CHARMM27 force field with CMAP corrections for proteins [36] and CHARMM36 lipid force field [37] in NAMD 2.7 [38] using the Nose-Hoover Langevin piston algorithm and PME for electrostatic interactions. LeuT_POPE/POPG_ was run under semi-isotropic pressure coupling conditions and LeuT_MNG-3_ was run under isotropic pressure coupling conditions. For more details, see Supporting Methods in File S1. The trajectories used for the analysis are from the production phase and only include the segment of the simulations after the Cα RMSD had converged. The total lengths of the equilibrated trajectories were 148 ns for LeuT_POPE/POPG_ and 146 ns for LeuT_MNG-3_.

### Definition of Functional Residue Clusters

**Figure 3:**
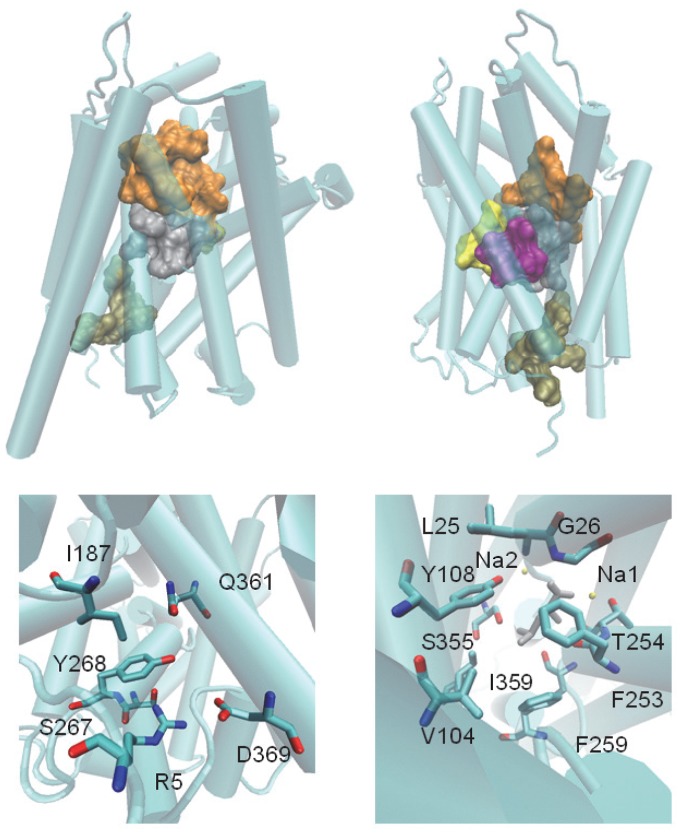
The structure of LeuT. Top panels: The 3GJD crystal structure of LeuT from two perspectives. TMs are displayed as cyan cylinders connected by loops. Each frc-site is represented by an outer surface: S1 (grey), S2 (orange), INI (tan), Na1 (yellow) and Na2 (purple). Bottom left: The INI-frc; numbers refer to the residue identity. Bottom right: The S1-frc (the leucine substrate is in grey, Na2 is added for reference).

Mechanistic and structure-function studies of LeuT as a prototypical NSS transporter have identified specific residues and structural microdomains that have significant roles in functional mechanisms. These include the binding sites for substrate and ions identified in the crystal structures [5], [10], [24], as well as the intracellular gate and surrounding interaction network, which has been shown to be involved in the transport mechanism [6]. We used these findings to define functional residue clusters (*frc*-s). Specifically, we defined the S1-frc to include the substrate, leucine, and residues L25, G26, V104, Y108, F253, T254, S256, F259, S355, and I359. The NA1-frc includes the bound ion, leucine, and residues A22, N27, T254, and N286 of the Na1 binding site. The NA2-frc is composed of the second ion bound, and residues G20, V23, A351, T354, and S355 of the Na2 binding site. We defined the S2-frc as composed of L29, R30, Y107, I111, W114, F253, A319, F320, F324, L400, and D404, and the intracellular gate region as an “intracellular network of interactions”, INI-frc, composed of R5, I187, S267, Y268, Q361, and D369. The locations of these sites in the LeuT structure are presented in Fig. 3.

### Post-Processing of the MD Trajectories

#### Accounting for symmetries

In order to estimate entropy from MD simulations, the coordinate of each atom is tracked throughout the trajectory to create a distribution of Cartesian coordinates. For side chains that display symmetry (Phe, Tyr, the carboyxlate groups of unprotonated Glu and Asp), simple tracking of atoms based on their numbering in the structure file can make symmetric states appear non-symmetric. To account for this, we used a clustering algorithm to group states by dihedral angles, and then divide the states by symmetry. For Phe and Tyr, we defined the state of the ring by the dihedral angle formed by the Cα, Cβ, the benzyl carbon bound to Cβ, and a benzyl carbon para to that carbon. For Glu and Asp, the state of the carboxylate was defined as the dihedral angle formed by N, Cα, the carbonyl carbon, and a carboxylate oxygen. For each residue, the *sin* and *cos* of each angle was calculated in order to project the angles onto the unit circle. Finally, the projections were collected into two clusters using the k-means clustering algorithm (implemented in R using the kmeans function in the stats package). If the angle between the centers of the two clusters was >90°, the position of the fourth atom was rotated by 180° relative to the plane formed by the first three atoms (as listed above) in frames from the second cluster.

#### Clustering of MD simulations

From analysis of a large number of LeuT simulations in our lab, we became aware of long-lived rearrangements in the conformation of the INI. Because the normal approximation we used for determining entropies may not be appropriate if there are large changes to the state of a set of residues, we determined first if there were distinct substates of the INI, by using k-means clustering on the minimum distances between side chains in the INI. Indeed, this revealed the transition between two long-lived states in the two simulations used for the NbIT analysis. Specifically, in LeuT_POPE/POPG_, the system transitioned after ∼118 ns from the crystal structure configuration in which R5 interacts with D369 and S267 in the INI, to a new configuration where R5 interacts with the surrounding water. In LeuT_MNG-3_, the equilibrated portion of the simulation begins with R5 interacting with the D369 and S267, but after ∼25 ns there is a transient rearrangement event, leading to a state in which R5 breaks away from D369, followed by a return of the INI to its original state after ∼20 ns. In order to isolate these states, MD simulation trajectories were clustered by the minimum distance between non-hydrogen side chain atoms of residues within the *frc*-s using the k-means clustering algorithm. Distance time series were smoothed over 1 ns windows to minimize thermal noise, and the best clustering was taken from 100 k-means runs. We performed the same clustering analysis using each *frc* individually, and found that not only did the INI have the most conformational variability (nearly an order of magnitude greater sum of square distance between frames in comparison to the other frc-s), but clustering into two states accounted for most of the variability (see Table S2 in File S1). Furthermore, we determined the similarity between results of clustering by the conformation of a specific *frc* versus all *frc*-s, by calculating the overlap as:
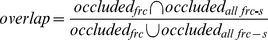
(1.1)

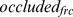
 corresponds to the set of frames in the occluded state when clustered by a given frc, whereas 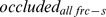 corresponds to the set when clustered by all *frc-s*. We find that clustering by all residues in the *frc*-s of interest provided a near identical result to clustering specifically by the INI. These results indicate that the INI rearrangement is the only significant rearrangement of a structural motif that takes place in the simulation trajectories. As the interaction between R5, D369, and S267 is observed crystallographically, we focused the study herein on comparing only this state from both simulations, in trajectories of over 100 ns from each simulation. While it might be interesting eventually to study as well the minor states of the INI not observed crystallographically, in which the gate is broken, these were not sampled sufficiently in either trajectory and thus are not yet adequate for rigorous analysis.

### Information Theory Quantities

#### Estimation of configurational entropy

In order to estimate the configurational entropies [39] from MD simulations, we first approximate the probability distribution of the atomic coordinates as a 3N-dimensional multivariate normal distribution of the multivariate random vector 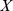, where 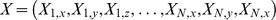 and 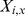, 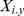, and 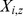 are the random variables corresponding to the x, y, and z coordinates of atom i, respectively. We then calculate the entropy analytically from the probability density function describing the distribution of 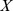. The probability distribution is defined as: 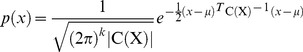
(2.1)

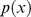
 is the probability density when 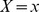 (i.e., when the multivariate random vector X has value *x*), 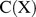 is the covariance matrix, 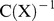 is the inverse of the covariance matrix, k is the rank of the covariance matrix, and μ is the vector of mean coordinates. In a Cartesian coordinate system, each covariance matrix can be estimated directly from the atomic fluctuations in the MD trajectory (the atomic fluctuation for a given frame, in a given coordinate axis, is the deviation the average coordinate in that axis). The covariance between variables 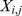 and 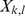, where i and k correspond to the atom index and j and l correspond to the dimension index, is calculated as: 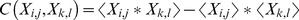
(3.1)


Covariances were calculated using **carma** [40]. The entropy of the continuous multivariate normal distribution can be calculated analytically through the differential entropy:
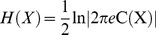
(4.1)

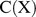
 is the covariance matrix describing all variables in 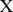. For each residue or set of residues, we consider all non-hydrogen atoms, and we apply here an approximation for the entropy that has been used recently [41]. This approximation is similar to previous harmonic [42] and quasi-harmonic [43] approximations, and we note that the calculations for the NbIT method are not limited to the use of any of these approximations, and can utilize other non-harmonic approximations of configurational entropy.

#### Mutual information

The mutual information between two residues and/or two sets of residues 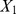 and 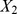 is the Kullback-Liebler divergence between the joint distribution and the product of the marginal distributions:
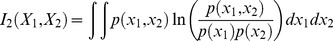
(5.1)

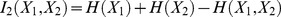
(5.2)


We use I_n_ to describe the information that is shared between all n bodies.

#### Co-information

3-body co-information is defined as:
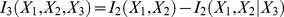
(6.1)

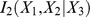
 is the *conditional mutual information* between 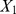 and 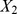, conditioning on 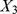:
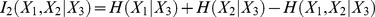
(7.1)

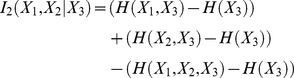
(7.2)


Co-information can be visualized easily using an information Venn diagram (see Fig. S3 in File S1). While several representations of this information are found in the literature with varying signs, we have chosen to use the sign convention described by [26], [29]. Using this convention, when co-information is positive, the third body may increase the information transmission between the two others, whereas when it is negative, the third body diminishes it.

In order to compare co-information, we calculate the normalized co-information defined by:
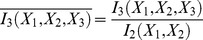
(8.1)where 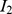 is the mutual information between the transmitter and receiver and 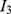 is the co-information between the transmitter, receiver, and channel. This measure is not equivalent for all possible assignments of 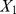, 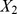, and 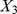 to transmitter, receiver, and channel.

#### Total correlation and coordination information

*Total correlation* (TC) describes the total amount of information that is shared among multivariate random variables in a set, and is a generalization of mutual information. TC is the Kullback-Liebler divergence between the product of the marginal distributions of the N multivariate random variables and the joint distribution.
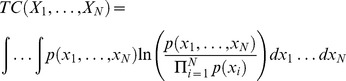
(9.1)

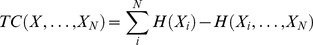
(9.2)


We generalize co-information to describe how much information that is shared by a set of variables of arbitrary size is also shared with another variable. This is calculated as the difference between the TC and the conditional TC, which we will call the *coordination information*:
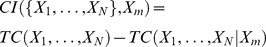
(10.1)

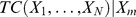
 is the *conditional total correlation* between 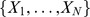, conditioning on 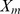: 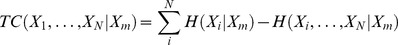
(11.1)

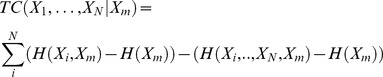
(11.2)


It should be noted that this generalization is not equivalent to the generalization of co-information to N-body information described by others previously [28]. Our generalization describes the amount of the total correlation in a set that is shared with another variable, and is only symmetric in the special case of a set of 2.

In order to compare coordination information, we calculate the *normalized coordination information*,
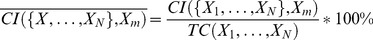
(12.1)


#### Coordination channel analysis

In order to define channels that mediate coordination information, we calculate the amount of coordination information that is shared between two residues and the same set, which we call *mutual coordination information,*

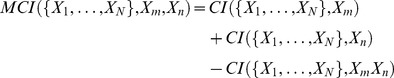
(13.1)


We then calculate the *normalized mutual coordination information*,
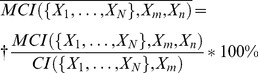
(14.1)


#### Calculation of single residue contributions to information measures

To identify residues that contribute significantly to information measures, we calculated the contribution of a single residue to an arbitrary information metric, I, as:
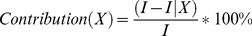
(15.1)


For details as to how this contribution was calculated for specific information measures, see Supplementary Methods in File S1.

We are currently in the process of creating an open-source R package that will be released to the community at a later date and will include the tools described here as well as additional tools that are in development. Information regarding resource packages is provided at http://physiology.med.cornell.edu/faculty/hweinstein/resources.html.

## Results

NbIT analysis was developed to provide unique insight into the molecular interactions driving global, coordinated motions, in the framework of information theory concepts developed for many-body systems. Thus, NbIT is ideally suited for analysis of biomolecular systems that display ligand-modulated coordinated motions in functional domains, as illustrated here for LeuT which serves this purpose well by virtue of its well-studied properties as an allosteric membrane protein that displays ligand-modulated dynamics. Importantly, the detailed molecular information available for LeuT from experimental and computational evaluations of structure-function relations in the intracellular gates, and the ion and substrate binding sites, makes it possible to probe directly the results from NbIT analysis. The MD trajectories analyzed with NbIT for this illustration of the method include only the long segments in which the interaction between R5, D369, and S267, which is observed crystallographically, is maintained (see above, section on “Clustering of MD Simulations”).

### The Pairwise Mutual Information

The analysis of pairwise *mutual information* for each of the functional residue clusters (*frc*-s) we defined (see “Methods: Defining Functional Residue Clusters”) in the crystallographically determined state, is summarized in Table 1. The calculated values show that the component residues in each of the *frc*-s exhibit coupled motions within the LeuT state studied here, as indicated by the mutual information that is greater than zero. Note, however, that it is difficult to compare the strength of coupling between two different sets of *frc*-s, because mutual information cannot be easily normalized from differential entropies calculated from multivariate normal distributions (see “Supporting Discussion 2: Normalizing Mutual Information” in File S1 for additional discussion). Therefore, we will not discuss further below the coupling strength between sites until we discuss other measures of information that can be normalized.

**Table 1:** Mutual information between known function sites in LeuT_POPE/POPG_.

	S1	S2	Na1	Na2	Na1, Na2	Na1, Na2, S1	Na1, Na2, S1, S2	INI
**S1**	***−328.1 (0.5)***	23.4 (0.6)	9.3 (0.1)	7.1(0.1)	13.2 (0.2)	X	X	12.9 (0.3)
**S2**	X	***−356.3 (0.7)***	14.9 (0.3)	7.5 (0.2)	21.6 (0.6)	33.0 (1.0)	X	15.1 (0.4)
**Na1**	X	X	***−141.2 (0.1)***	8.3 (0.1)	X	X	X	4.8 (0.1)
**Na2**	X	X	X	***−112.9*** ** (0.1)**	X	X	X	4.0 (0.1)
**Na1, Na2**	X	X	X	X	***−262.4 (0.12***	X	X	8.4 (0.3)
**Na1, Na2, S1**	X	X	X	X	X	***−519.2 (0.9)***	X	18.1 (0.6)
**Na1, Na2, S1, S2**	X	X	X	X	X	X	***−869.6(2.5)***	31.7 (1.2)
**INI**	X	X	X	X	X	X	X	***−136.8 (1.4)***

Off-diagonal elements correspond to the mutual information between two given *frc-s*, where as the diagonal elements correspond to the entropy of a given *frc*. Units are in nats.

### The Communication Channel Coupling the S1-frc to the INI-frc Utilizes TM6

A central mechanistic question regarding the functional dynamics of transporters is how the binding of substrate can trigger the conformational reorganization leading to the intracellular-open state from which the substrate is eventually released. Because studies have shown that just the binding of Na^+^ and substrate cause measurable dynamic effects at the intracellular end of the LeuT molecule, even in the absence of transport [7], [8], we sought to determine the information channel enabling this allosteric behavior. To this end, we performed *co-information analysis* as described in Methods to evaluate which residues played the role of channel in the information exchange between the substrate sites and the INI.

As described, the co-information describes the information shared between all residues in a set. We calculated the 3-body co-information between each *frc* and a potential single residue channel using Equation 6.1 and then normalized as to the mutual information between the sites (see “Co-information” in Methods) to determine how much of the allosteric coupling could be attributed to that residue. In the interpretation of these results we considered that in a simple transmitter-channel-receiver system, the 3-body co-information can be understood intuitively as the intersect of the three entropies in a 3-body information Venn diagram (see Fig. S2 in File S1), and can determine how much of the mutual information between the receiver and transmitter can be explained by the information they both share with the channel.

The calculated values are shown as a *co-information* versus *co-information rank* plot, which features a linear middle region with high, and low, co-information extremes (see Fig. S4 in File S1). Based on the plot, we defined residues to be potential *channels* if they were in the region of the high co-information extreme (see “Supporting Methods: Identifying High Co-Information Residues” in File S1). We note that the criterion of high co-information is not sufficient to differentiate between a true channel and a residue that has high mutual information with a true channel. However, the latter will display lower co-information than the former, and thus our most confident channel predictions are the residues with the highest co-information as described below (for an illustrative example using a model system, see “Supporting Discussion: Analysis of the K1,4 Network” and Fig. S4 in File S1).

**Figure 4:**
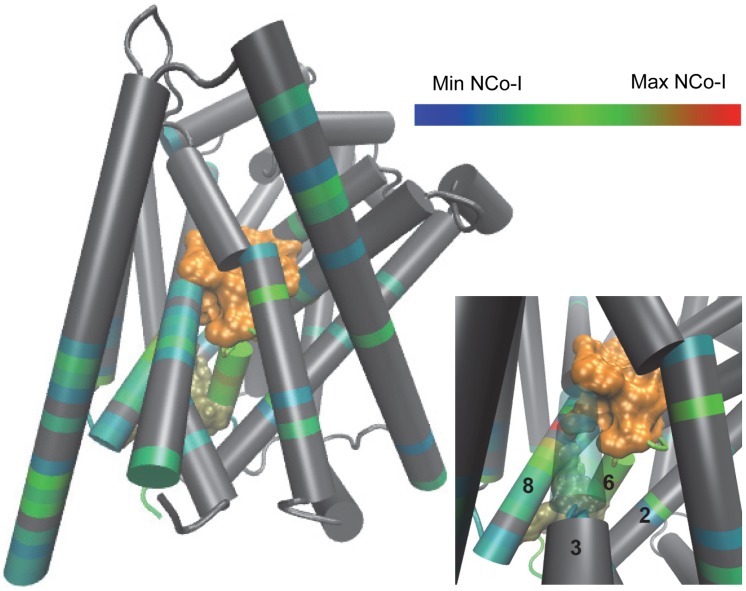
TMs 2, 6b, and 8 form a co-information channel between S1 and the INI in LeuT_POPE/POPG_. Main: Residues found to have high co-information with S1 and the INI are colored by normalized co-information (NCoI) values using the scale at the top right (range from smallest-in blue to largest-in red); Min and Max NCoI refers to the minimum and maximum values among all possible residues. S1 is in orange surface and the INI is in tan surface; all other residues are represented in grey. Bottom right: A close up of the TM2, TM6b, and TM8 interface. The definitions of the S1 and INI frc-s can be found in **Methods**.

Applying co-information analysis reveals that S1 and the INI are coupled through a set of residues consisting largely of residues from TM6b, TM8, and TM2 (See Fig. 4). Co-information analysis also reveals a channel between S2 and the INI, which is similarly composed of residues from TM6b and TM8, in addition to residues from S1 in the unstructured region between TM6a and TM6b (see Fig. S5 in File S1).

Not all the residues in a particular *frc* contribute equally to the allosteric communication. In order to identify which residues within the substrate sites and the INI are essential for allosteric communication we identified the residues within these sites that made large contributions to the mutual information. Such residues contribute by coupling the sites directly to the channel, and by distributing the information throughout the rest of their respective site. They were identified from the calculated values of their contribution to the mutual information, expressed as the percentage of the *mutual information* that could be explained **by conditioning on that residue** (see Methods, “Calculation of Single Residue Contributions to Information Measures”). Calculated in this manner, the percentage of the *mutual information* describes how much of the information shared between the two sites is shared with that residue specifically. It is essential to note that the total sum of contribution from all residues does not necessarily sum to 100%. This occurs because just as the residues share information, they can also share their contribution to the mutual information, so the sum of the contribution will exceed 100%. This is also the case for other contribution measures, as described further below.

Using Equation S.1 in File S1, we found that for the coupling between the S1-frc and the INI, it is residues I359, F259, F253 in the S1-frc that make the largest contributions (21.2% 18.8%, and 12.5% respectively), and in the INI the largest contribution is from residues Q361, R5, and Y268 (28.3, 21.6%, and 21.3% respectively%). These very specific identifications underscore the validity of the calculated communication channel, as they are consistent with results from previous work in which mutations of I359 and F259 were shown to modulate transport efficacy [44]. Interestingly, we find that for the coupling between the S2-frc and the INI, residues R30, F324, and W114 make the largest contributions in S2 (20.1%, 12.9%, and 12.5%), and in the INI residues R5, I187, and Y268 make the largest contributions (27.1%, 23.3%, and 9.5% respectively). Because R30 is considered to form an extracellular gate with D404, the significant role we find for it here in the coupling of S2 and the INI underscores the strong relationship between the extracellular and intracellular gates. These results are summarized in Table 2 and 3.

**Table 2:** Specific residues highly contribute to mutual information between S1 and the INI in LeuT_POPE/POPG_.

**S1**	**Leu**	**L25**	**G26**	**V104**	**Y108**	**F253**
	10.5% (0.1%)	9.9% (0.0%)	6.4% (0.0%)	8.4% (0.1%)	11.8% (0.1%)	**12.5% (0.1%)**
	**T254**	**S256**	**F259**	**S355**	**I359**	**Na1**
	8.8% (0.1%)	9.3% (0.1%)	**18.8% (0.1%)**	7.7% (0.1%)	**21.2% (0.2%)**	3.0% (0.0%)
**INI**	**R5**	**I187**	**S267**	**Y268**	**Q361**	**D369**
	**21.6% (0.3%)**	19.7% (0.4%)	14.6% (0.1%)	**21.3% (0.1%)**	**28.3% (0.3%)**	15.6% (0.1%)

The contribution of specific residues in S1 (top) and the INI (bottom) to the communication between S1 and the INI (top 3 in each site are bold).

**Table 3:** Specific residues highly contribute to mutual information between S2and the INI in LeuT_POPE/POPG_.

**S2**	**L29**	**R30**	**Y107**	**I111**	**W114**	**F253**
	8.8% (0.6%)	**20.1% (0.0%)**	9.9% (0.1%)	7.5% (0.1%)	**12.5% (0.1%)**	10.6% (0.1%)
	**A319**	**F320**	**F324**	**L400**	**D404**	
	6.1% (0.1%)	10.2% (0.1%)	**12.9% (0.1%)**	9.1% (0.1%)	8.6% (0.1%)	
**INI**	**R5**	**I187**	**S267**	**Y268**	**Q361**	**D369**
	**27.1% (0.3%)**	**23.3% (0.5%)**	14.6% (0.2%)	**19.5% (0.1%)**	17.3% (0.2%)	18.2% (0.2%)

The contribution of specific residues in S2 (top) and the INI (bottom) to the communication between S2 and the INI (top 3 in each site are bold).

### The Coordination within frc-s Is Performed by Known Functional Residues

We hypothesized that that the proper fold and specific local function of a given *frc*, such as substrate binding, are maintained through short-distance allosteric couplings underlying collective behavior among the residues in the clusters. We probed this by calculating the *total correlation* (TC) for each *frc* to obtain a measure of the total amount of information shared by a set of size *N* through any type of correlation from 2 to *N*-body. We then calculated the contribution of a given residue in the *frc* to this TC (see Methods, “Total Correlation and Coordination Information”).

With this approach, we find that in the INI, the three largest contributors are Y268 (60.7%), S267 (59.0%) and R5 (42.7%). This is consistent with their central location in the INI topology and with previous reports that mutation of the highly conserved Y268 and R5 to alanine has a strong effect on the structure and dynamics of the intracellular gate [6], [7]. In the S1-frc, the largest contributions to the TC were calculated to come from T254 (40.3%), the leucine substrate (38.9%), and F253 (38.9%). The bound Leu is expected to contribute strongly, as seen here, because it interacts with all other residues in S1. Furthermore, as mutation of F253 has been shown to greatly reduce binding in S1 [8], [45], it is possible that its role is not only to stabilize Leu binding through direct interaction, but also to stabilize the site as a whole by coordinating the rest of the S1 residues.

In the other *frc*-s we also found a small number of specific high contributions. Thus, in the Na1 site the largest contributions to the total correlation are made by the Na1 sodium ion (61.7%), T254 (60.1%), and by leucine (58.4%). Interestingly, in the Na2 site, T354 and S355 contribute significantly more (70.9% and 66.4%, respectively) than the Na^+^ ion (52.1%). Finally, in S2, residues F320, A319, and R30 are found to make the largest contributions of 39.6%, 33.0%, and 31.1%, respectively. These results are summarized in Table 4.

**Table 4:** The contribution of specific residues to the total correlation of their sites in LeuT_POPE/POPG_.

**S1**	**Leu**	**L25**	**G26**	**V104**	**Y108**	**F253**
	**38.9% (0.2%)**	36.2% (0.2%)	32.3% (0.3%)	13.2% (0.1%)	23.3% (0.1%)	**38.9% (0.2%)**
	**T254**	**S256**	**F259**	**S355**	**I359**	**Na1**
	**40.3% (0.3%)**	29.1% (0.2%)	20.1% (0.2%)	13.6% (0.2%)	12.1% (0.1%)	20.2% (0.2%)
**S2**	**L29**	**R30**	**Y107**	**I111**	**W114**	**F253**
	25.6% (0.1%)	**31.1% (0.2%)**	17.4% (0.1%)	17.4% (0.1%)	18.5% (0.1%)	10.9% (0.0%)
	**A319**	**F320**	**F324**	**L400**	**D404**	
	**33.0% (0.3%)**	**39.6% (0.3%)**	20.9% (0.1%)	14.0% (0.1%)	15.0% (0.1%)	
**Na1**	**Na1**	**A22**	**N27**	**T254**	**N286**	**Leu**
	**61.7% (0.3%)**	49.5% (0.2%)	50.0% (0.2%)	**60.1% (0.2%)**	36.3% (0.2%)	**58.4% (0.2%)**
**Na2**	**Na2**	**G20**	**V23**	**A351**	**T354**	**S355**
	**52.1% (0.2%)**	37.6% (0.2%)	40.1% (0.2%)	38.6% (0.2%)	**70.9% (0.1%)**	**66.4% (0.1%)**
**INI**	**R5**	**I187**	**S267**	**Y268**	**Q361**	**D369**
	**42.7% (0.4%)**	34.8% (0.8%)	**59.0% (0.6%)**	**60.7% (0.4%)**	23.8% (0.4%)	28.8% (0.4%)

For each *frc*, the contribution of each residue to the total correlation is presented. The top 3 residues in each site are shown in bold.

### Both the S1-frc and the S2-frc Coordinate Multi-Body Collective Motions in the INI

Key findings from smFRET experiments investigating the allosteric modulation of intracellular gating in LeuT [7] were that conformational changes in the intracellular gates require collective motions resulting in large spatial displacements, and that these motions are modulated (in some undetermined way) by the state of the substrate binding sites, S1 and S2 [8]. In order to investigate the role of these substrate binding sites in the collective dynamics within the INI-frc, we calculated how much each of the two binding sites contributed to the total correlation of INI. This contribution, termed here *coordination information* (CI), describes the amount of total correlation in a set of variables (the “coordinated set”, here the INI-frc) that is shared with a variable (or multivariate distribution) that is not included in the coordinated set (“the coordinator”, here the S1 or S2 frc-s) (see Methods, “Total Correlation” and “Coordination Information”, and Fig. S6 in File S1). When calculated in this manner, CI describes the contribution of a site to all possible n-body correlations within another site (for an illustrative example using a model system, see “Supporting Discussion: Analysis of the K1,4 Network” in File S1). Here we used as the descriptor the *normalized coordination information* (NCI), in which the coordination information is normalized to the total correlation within the coordinated site. It should be noted that *coordinators* are not all *coordination channels*. *Coordinators* can be coupled to *coordination channels*, and thus perturbation to the *coordinator* leads to a perturbation in the coordinated set.

As summarized in Table 5, the NCI calculated for S1 and S2 show that they both coordinate the INI, with values of 19.1% for S1, and 21.2% for S2. The Na1 and Na2 sites coordinate the INI only weakly (NCI = 9.0% and 6.9%, respectively), and their combined NCI in coordinating the INI is 11.1%. The coordination of INI by the combination of S1, S2, and the Na1 and Na2 frc-s is 27.1%, indicating that just under a third of all the correlated motions in the INI are related to these sites. The coordination exerted by INI on the binding sites was also calculated, because coordination information is not symmetric. We find that while S1 and S2 coordinate the INI strongly, the INI coordinates the two only moderately (NCI = 12.0% and 7.4%, respectively). Interestingly, in the MD trajectory we analyzed, the coordination by INI of the Na1 (NCI = 14.2%) and Na2 (NCI = 10.5%) sites is stronger than in the opposite direction. These results, along with results for all comparisons of sites, are summarized in Table 5. To estimate the importance of these coordination values for the allosteric mechanism, we performed control calculations of the normalized coordination information for S1 and S2, with several other intracellular sites not known for their functional roles, including specific helices, loops, and interfaces between them. In all cases, S1 and S2 coordination of any of these control sites was half (or much less) that of the INI (see “Supplementary Results: Coordination of Other Intracellular Domains”, Fig. S8, and Table S1 in File S1).

**Table 5:** Normalized coordination information between sites in LeuT_POPE/POPG_.

	S1	S2	Na1	Na2	Na1, Na2	Na1, Na2, S1	Na1, Na2, S1, S2	INI
**S1**	***30.6 (0.2)***	23.8% (0.5%)	27.5% (0.4%)	17.3% (0.3%)	31.0% (0.5%)	X	X	12.0% (0.4%)
**S2**	14.2% (0.4%)	***33.1*** ** (0.4)**	8.2% (0.2%)	4.5% (0.2%)	8.9% (0.3%)	15.0% (0.5%)	X	7.4% (0.3%)
**Na1**	51.5% (0.5%)	44.2% (0.5%)	***9.2*** ** (0.1)**	39.1% (0.3%)	X	X	X	14.2% (0.5%)
**Na2**	40.1% (0.4%)	16.7% (0.3%)	32.4% (0.3%)	***12.04*** **(0.1)**	X	X	X	10.5% (0.3%)
**Na1, Na2**	32.1% (0.3%)	23.3% (0.4%)	X	X	***29.8*** ** (0.2)**	X	X	10.1% (0.3%)
**Na1, Na2, S1**	X	16.3% (0.5%)	X	X	X	***67.2*** ** (0.6)**	X	8.8% (0.3%)
**Na1, Na2, S1, S2**	X	X	X	X	X	X	***132.9*** ** (2.0)**	6.2% (0.4%)
**INI**	19.1% (0.6%)	21.2% (0.7%)	9.0% (0.3%)	6.9% (0.3%)	11.1% (0.4%)	20.5% (0.7%)	27.1% (1.2%)	***14.3*** ** (0.1)**

For each pair of *frc-s*, the normalized coordination information is presented, with residues on the top (columns) acting as the coordinator and residues on the left (rows) being coordinated. On the diagonal, the total correlation of the site is shown in bold.

Given the importance of the INI in the function of the transporter, we also determined which individual residues make the largest contributions to coordination of the INI. For each residue in the S1-frc and S2-frc residue we calculated the contribution of the residue to the particular frc coordination of the INI, as well as the contribution of INI residues to receiving that coordination, using Equation S.3 in File S1. Results summarized in Table 6 show that for coordination of the INI-frc by S1, the top 3 *coordinators* are F259 (contribution = 69.6%), S256 (contribution = 34.9%), and I359 (contribution = 34.6%), and the top 3 *receivers* are R5 (contribution = 67.8%), I187 (contribution = 63.8%), and S267 (contribution = 59.9%). For coordination by S2 (see Table 7), the top 3 *coordinators* are R30 (contribution = 54.7%), F253 (contribution = 28.7%), and F324 (contribution = 24.0%), and the top 3 *receivers* are R5 (contribution = 80.8%), I187 (contribution = 71.0%), and D369 (contribution = 58.1%). This underscores the important role of INI residues R5, I187, and S267 in the coordination of the INI-frc by the known allosteric substrate sites.

**Table 6:** Specific residues highly contribute to coordination of the INI by S1 in LeuT_POPE/POPG_.

**S1**	**Leu**	**L25**	**G26**	**V104**	**Y108**	**F253**
	24.5% (0.1%)	22.8% (0.2%)	21.9% (0.2%)	18.8% (0.2%)	13.9% (0.1%)	31.0% (0.2%)
	**T254**	**S256**	**F259**	**S355**	**I359**	**Na1**
	27.3% (0.2%)	**33.6%** (0.3%)	**67.6%** (0.2%)	13.3% (0.2%)	**33.2%** (0.4%)	16.6% (0.1%)
**INI**	**R5**	**I187**	**S267**	**Y268**	**Q361**	**D369**
	**66.1%** (0.3%)	**63.1%** (0.2%)	**58.7%** (0.1%)	57.3% (0.2%)	57.2% (0.4%)	48.1% (0.3%)

The contribution of specific residues in the S1-frc (top) and the INI-frc (bottom) to the coordination of the INI-frc by the S1-frc (top 3 in each site are bold).

**Table 7:** Specific residues highly contribute to coordination of the INI by S2 in LeuT_POPE/POPG_.

**S2**	**L29**	**R30**	**Y107**	**I111**	**W114**	**F253**
	20.1% (0.2%)	**53.8% (0.5%)**	10.6% (0.2%)	9.0% (0.3%)	14.6% (0.2%)	**28.0% (0.2%)**
	**A319**	**F320**	**F324**	**L400**	**D404**	
	10.7% (0.0%)	11.4% (0.1%)	**23.2% (0.1%)**	16.2% (0.1%)	18.8% (0.1%)	
**INI**	**R5**	**I187**	**S267**	**Y268**	**Q361**	**D369**
	**78.3% (0.2%)**	**69.0% (0.2%)**	48.5% (0.2%)	42.5% (0.3%)	40.0% (0.3%)	**57.6% (0.4%)**

The contribution of specific residues in the S2-frc (top) and the INI-frc (bottom) to the coordination of the INI-frc by the S2-frc (top 3 in each site are bold).

### The Coordination Channel Mediating the INI-frc Coordination by the Substrate *frc*-s Is through TM6b

Because TM6b emerged as the major channel for communication between S1 and the INI, we investigated whether it was also the major channel for the CI between the substrate sites and the INI. We calculated the *mutual coordination information* (MCI) using Equation 13.1, which described how much of the coordination information is shared between two coordinators that are coordinating the same set (see Methods, Coordination Channel Analysis), and then normalized to the coordination information of the coordinator of interest (NMCI). Using this analysis, we identified residues in the high NMCI region using the same criteria described for *co-information*. The results identify a *coordination channel* that is nearly identical to the channel revealed by the *co-information analysis*, with a significantly larger signal in TM6b than that calculated with co-information analysis (see Fig. 4). We are able to identify a similar coordination channel for S2 (see Fig. S9 in File S1). These results indicate that TM6b is the major channel for the coordination of the INI by S1 and S2.

### The Allosteric Couplings Calculated for LeuT in MNG-3 Micelles Are Similar to Those in Membranes

**Figure 5:**
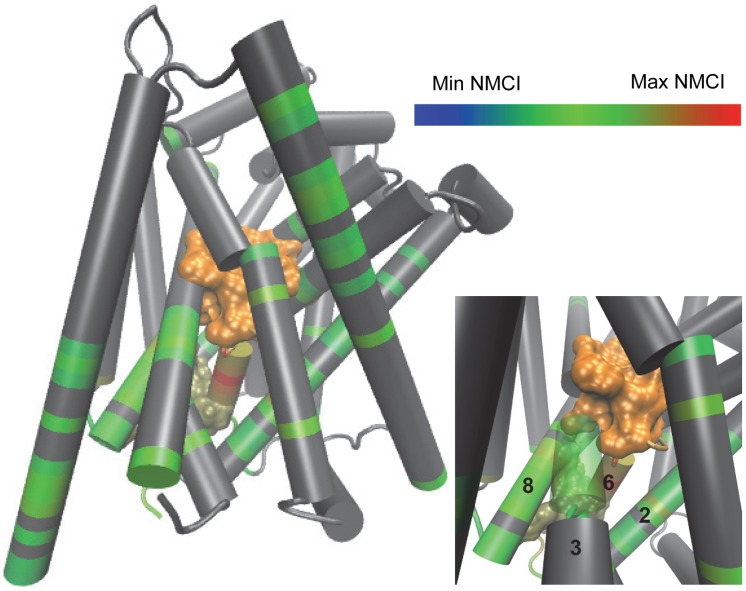
TMs 2, 6b, and 8 form a coordination channel between S1 and the INI in LeuT_POPE/POPG_. Main: Residues found to have high mutual coordination information with S1 and the INI are colored by normalized mutual coordination information (NCMI) using the scale at the top right, where the minimum and maximum NCMI refers to the minimum and maximum among all possible residues. S1 is in orange surface and the INI is in tan surface; all other residues are represented in grey. Bottom right: A close up of the TM2, TM6b, and TM8 interface. The definitions of the S1 and INI frc-s can be found in **Methods**.

Detergent micelles are a common environment used in experimental studies of membrane proteins e.g., crystallography and biophysical experiments such as isothermal calorimetry and smFRET. Previous work has indicated that some detergents may affect measurements such as binding affinity and stoichiometry [24], [46], [47]. Here we investigated the same LeuT construct examined by simulations in membranes, in a micellar environment composed of MNG-3 detergent, which has been shown not to have the same detrimental effects as other detergents in several experimental measurements of LeuT [48]. Our findings agree, as the allosteric coupling measures calculated for LeuT_MNG-3_ are comparable to those we obtained for LeuT_POPE/POPG_ (see Table S3 in File S1 for LeuT_MNG-3_ and Table 5 for LeuT_POPE/POPG_), albeit with some noticeable changes to allosteric couplings involving only the Na^+^ sites. Despite these changes, the contribution of specific residues to the total correlation of their *frc* remains conserved, and so do the major contributors to the total correlation (see Table S4 in File S1 for LeuT_MNG-3_ and Table 4 for LeuT_POPE/POPG_). In addition, the major contributors to coordination between the substrate site *frc*-s and the INI are also preserved (see Table S5 in File S1 for LeuT_MNG-3_ and Table 6 for LeuT_POPE/POPG_), and together the results for LeuT_MNG-3_ indicate that the allosteric behavior seen in the membrane simulation is conserved in the micelle simulation. It is worth noting however, that in the LeuT_MNG-3_ the coordination channel between the S1 and the INI *frc*-s includes fewer residues than in LeuT_POPE/POPG_, although they are still mainly from TM6b (see Fig. S10 in File S1 for LeuT_MNG-3_ and Fig. 5 for LeuT_POPE/POPG_), but so few residues are identified for coordination by S2 (see Fig. S11 in File S1 for LeuT_MNG-3_ and Fig. S9 in File S1 for LeuT_POPE/POPG_) that a clear coordination channel is not resolvable between S2 and the INI in LeuT_MNG-3_. In an additional analysis suggested in the review process, we compared these results to those obtained from an apo (substrate-free) state of LeuT, by analyzing a trajectory (see “Supporting Methods: MD Simulations” in File S1 for details) provided by Dr. Lei Shi (data unpublished, personal communication). Again, we find TM6b to be the major channel for coordination of the INI by both S1 and S2.

## Discussion

Taking advantage of the information about specific functional motifs for the allosteric transporter LeuT, the illustration of the new NbIT analysis method brings to light how it identifies the details of allosteric couplings, and can quantify them at a previously unattained level of detail. Moreover, the choice of LeuT for this illustration of NbIT allowed us not only to start from well-defined *frc*-s, but also to compare the results and the inferences from NbIT analysis to known mechanistic elements in the allosteric process underlying LeuT function. Indeed, the allosteric pathway between the known ligand (ions, substrate) binding sites and previously proposed functional elements such as the intracellular gate (in INI), were identified by the NbIT analysis as the channels that propagate these couplings. This agreement with previous mechanistic insights is important because computational approaches, and in particular the type of MD simulations utilized here as well, have been used successfully to study the dynamics of transporter molecules and to infer on residues and motifs that play essential roles in the allosteric mechanisms [13], [49]–[51], By taking advantage of this kind of data, the novel NbIT analysis provides the first rigorous method for the identification of specific channels by which information is transmitted between functional sites of an allosteric molecular system. Key observations from the present application of NbIT analysis are discussed below to stress the specific molecular detail of the results, and to indicate the predictive power that this new method can bring to the many other allosteric protein systems for which the type of information available for LeuT is currently lacking.

### 

#### 1. Allosteric coordination of the INI by S1 and S2

The CI calculations were essential in revealing that the S1 and S2 sites coordinate the internal dynamics of the INI (Table 5). The allosteric modulation of the intracellular gate considered on the single molecule macro scale (as described in the Introduction) has been noted previously in the dynamic changes revealed by smFRET experiments with LeuT in detergent; this study showed how the allosteric connection enabling modulation at the micro scale is effectuated. *Coordination information* as calculated here connects the collective coordination of the INI domain to the individual components (specific residues) and interactions (within, and outside the *frc* to which they belong) that underlie it. This provides insight at unprecedented detail about the elaborate coordination in the allosteric mechanism underlying ligand-induced opening of the gate. An intriguing observation in view of the ongoing controversy surrounding the role of the S2 binding site [11]–[13], [24], [46]–[48], [52] is that the S2-frc coordinates the INI through a channel that includes the S1 site (Table 4, Figure S6 in File S1). The coordination found here, of the INI by the *apo* S2 site (the MD trajectories analyzed here did not include substrate bound in S2) may explain why mutations to the S2 site have been shown to affect intracellular gating dynamics [7]. Although they demonstrate the ability of the S2-frc to coordinate the intracellular gate, the present results cannot inform about the role of substrate binding in S2 in the transport process, since this was not covered in the MD simulation.

#### 2. Propagation of information between S1, S2, and the INI requires TM6b

The channel that propagates the coordination of the INI by S1 and S2 was found here to consist largely of residues in TM6b (Fig. 5). Indeed, several residues in the S1 site and the INI are part of the highly conserved TM6, and its intracellular end, TM6b, was shown to undergo a large rotation of 17° in a recent crystal structure of a LeuT mutant stabilized in what is believed to be an *apo* intracellular-open state [53]; TM1a and TM8 also contain many residues from S1 and the INI.

Notably, while this manuscript was in preparation, a set of LeuT mutants have been described that were constructed to resemble the human serotonin transporter [54], and all constructs containing a mutation of the TM6b residue Y265 to F, were found to lack transport activity despite retaining high affinity inhibitor binding. This indicates a possible role of TM6b in function, and we interpret the observed rotation of TM6b and the effect of the Y265F mutation as support for their role in propagating information from the substrate site to the intracellular gate during the transition between LeuT states. The fact that the role of TM6b became evident from the NbIT analysis of the S1-occupied occluded state supports its role as an information conduit from the substrate sites to the intracellular gate.

#### 3. The intramolecular allosteric mechanism involves a subset of residues known to have functional roles

With NbIT analysis, we identified specific residues that play a role in allosteric connections related to function, and were able to discern different contributions (i.e., “stabilizers” and “communicators”). In the S1-frc we find that while the bound leucine substrate, F253, and T254 coordinate the binding site's internal correlations (hence acting as stabilizers), residues F259, S256, and Q359 contribute to the coupling between S1 and the INI (Table 5A) and belong to “communicators”, which are involved in between-site allosteric communication. We know of no previous computational method that offered such functionally specific discrimination.

The identification of functional roles for specific residues in the allosteric communication revealed further details of their mechanistic involvement:

Our analysis predicted that F259 interactions may have a significant effect on transport. Earlier crystallographic studies had indicates that F259 may be involved in the diversity of transport phenotypes produced by various LeuT substrates [55]. Three basic modes of interaction have been observed: (i)-in crystal structures of LeuT in complex with leucine, methionine, or p-flurophenylalanine, the hydrophobic side chains interact with F259; (ii)-in LeuT structures with alanine or glycine, this interaction is lost, leading to a 30° rotation of the F259 side chain; (iii)-in the structure bound to tryptophan, the indole ring makes a ring-ring contact with the F259 side chain. The three distinct modes of interaction observed for F259 correlate with distinct transport phenotypes. Thus, although the overall binding modes could appear nearly identical, the transport efficiencies differ, with alanine being transported with highest efficiency (k_cat_/K_m_); leucine, methionine, and p-flurophenylalanine displaying low efficiency, and tryptophan acting as an inhibitor. While the efficiency for glycine is even lower than for the low efficiency amino acids mentioned above, the difference may in fact be due to the very low affinity of Gly for LeuT which may not allow it to remain bound to the transporter long enough to initiate transport (no k_on_ or k_off_ values have been reported). Together, these structure/function relations suggest that substrate interactions with F259 may lead to different effects on transport. Our analysis predicted a specific participation in the allosteric mechanism. We suggest that because alanine does not interact with F259 and induces a change in the rotameric state of F259 relative to that observed for the less efficiently transported substrates, F259 plays an inhibitory role by allosterically blocking transport. Clarification of the specific role that this type of allosteric modulation plays in the transport cycle with the NbIT method must await a complete trajectory of the transition among the different states, but the insights gained in this study offer an intriguing avenue for future experimentation.

We find that Y268 R5, and S267 all play the role of both strong stabilizers and communicators in the INI. Both R5 and Y268 are known to be involved in function, with mutation of either residue to alanine resulting in disruption of the intracellular gate [6], [7], characterized by an increased “open” (intracellular gate) population observed in smFRET experiments of the intracellular gate. However, the R5A mutation has also been shown to cause increased transitions between the “open” and “closed” (intracellular gate) state in the presence of leucine [7]. Considered together, these experimental findings indicate that mutation of R5 can affect the allosterically modulated gating dynamics; in agreement, R5 is predicted to be the strongest *coordinator* within the INI. The result that Y268, S267, and R5 all play the role of both *coordinator* and *stabilizer* is especially noteworthy because one would expect that residues that are essential to the stability of the gate would need to be modulated in order to initiate large collective conformational changes, such as the opening of the gate. That such residues are also communicators substantiates the allosteric modulation of the conformational change that opens the gate. Indeed, these residues are highly conserved in NSS transporters [56], and our finding leads to the prediction that disruption of interactions between S267 and its surrounding network will strongly affect transport. Future experiments should be able to better define the role of S267 in the transport function based on this testable hypothesis. In addition, we find that while I187 has a minor stabilizer role in the INI, it plays a significant role as a communicator. This leads to the mechanistic prediction that mutation of I187 may lead to disruption of allosteric modulation without disrupting the structure of the intracellular gate.

## Supporting Information

File S1 **Supporting Methods (MD simulations; Moving block bootstrapping of MD simulations; Contributions for specific information measures; Identifying high information residues); Supporting Discussion (Efficient information transmission; Normalizing mutual information; Negative** ***co-information*** **; Analysis of the K_1,4_ network; Control study); Tables S1–S4; Figures S1–S11; Supporting References.**
(PDF)

## References

[1] Monod J, Changeux J-P, Jacob F (1963) Allosteric proteins and cellular control systems. J Mol Biol 6: 306–329 10.1016/S0022-2836(63)80091-1 13936070

[2] Gunasekaran K, Ma B, Nussinov R (2004) Is allostery an intrinsic property of all dynamic proteins? Proteins 57: 433–443 10.1002/prot.20232 15382234

[3] Jardetzky O (1966) Simple allosteric model for membrane pumps. Nature 969–970 10.1038/211969a0 5968307

[4] Krishnamurthy H, Gouaux E (2012) X-ray structures of LeuT in substrate-free outward-open and apo inward-open states. Nature 481: 469–474 10.1038/nature10737 22230955 PMC3306218

[5] Yamashita A, Singh SK, Kawate T, Jin Y, Gouaux E (2005) Crystal structure of a bacterial homologue of Na+/Cl–dependent neurotransmitter transporters. Nature 437: 215–223 10.1038/nature03978 16041361

[6] Kniazeff J, Shi L, Loland CJ, Javitch Ja, Weinstein H, et al (2008) An intracellular interaction network regulates conformational transitions in the dopamine transporter. J Biol Chem 283: 17691–17701 10.1074/jbc.M800475200 18426798 PMC2427322

[7] Zhao Y, Terry D, Shi L, Weinstein H, Blanchard SC, et al (2010) Single-molecule dynamics of gating in a neurotransmitter transporter homologue. Nature 465: 188–193 10.1038/nature09057 20463731 PMC2940119

[8] Zhao Y, Terry DS, Shi L, Quick M, Weinstein H, et al (2011) Substrate-modulated gating dynamics in a Na+-coupled neurotransmitter transporter homologue. Nature 474: 109–113 10.1038/nature09971 21516104 PMC3178346

[9] Andersen J, Taboureau O, Hansen KB, Olsen L, Egebjerg J, et al (2009) Location of the antidepressant binding site in the serotonin transporter: importance of Ser-438 in recognition of citalopram and tricyclic antidepressants. J Biol Chem 284: 10276–10284 10.1074/jbc.M806907200 19213730 PMC2665081

[10] Singh SK, Yamashita A, Gouaux E (2007) Antidepressant binding site in a bacterial homologue of neurotransmitter transporters. Nature 448: 952–956 10.1038/nature06038 17687333

[11] Shi L, Quick M, Zhao Y, Weinstein H, Javitch Ja (2008) The mechanism of a neurotransmitter∶sodium symporter–inward release of Na+ and substrate is triggered by substrate in a second binding site. Mol Cell 30: 667–677 10.1016/j.molcel.2008.05.008 18570870 PMC2826427

[12] Shan J, Javitch Ja, Shi L, Weinstein H (2011) The substrate-driven transition to an inward-facing conformation in the functional mechanism of the dopamine transporter. PLoS One 6: e16350 10.1371/journal.pone.0016350 21298009 PMC3029329

[13] Cheng MH, Bahar I (2013) Coupled Global and Local Changes Direct Substrate Translocation by Neurotransmitter-Sodium Symporter Ortholog LeuT. Biophys J 105: 630–639 10.1016/j.bpj.2013.06.032 23931311 PMC3736663

[14] Gleitsman KR, Shanata JaP, Frazier SJ, Lester Ha, Dougherty Da (2009) Long-range coupling in an allosteric receptor revealed by mutant cycle analysis. Biophys J 96: 3168–3178 10.1016/j.bpj.2008.12.3949 19383461 PMC2718292

[15] Sethi A, Eargle J, Black Aa, Luthey-Schulten Z (2009) Dynamical networks in tRNA:protein complexes. PNAS 106: 6620–6625 10.1073/pnas.0810961106 19351898 PMC2672494

[16] Chennubhotla C, Bahar I (2007) Signal propagation in proteins and relation to equilibrium fluctuations. PLoS Comput Biol 3: 1716–1726 10.1371/journal.pcbi.0030172 17892319 PMC1988854

[17] Del Sol A, Fujihashi H, Amoros D, Nussinov R (2006) Residues crucial for maintaining short paths in network communication mediate signaling in proteins. Mol Syst Biol 2: 2006.0019 10.1038/msb4100063 PMC1681495 16738564

[18] Amitai G, Shemesh A, Sitbon E, Shklar M, Netanely D, et al (2004) Network analysis of protein structures identifies functional residues. J Mol Biol 344: 1135–1146 10.1016/j.jmb.2004.10.055 15544817

[19] De Ruvo M, Giuliani A, Paci P, Santoni D, Di Paola L (2012) Shedding light on protein-ligand binding by graph theory: The topological nature of allostery. Biophys Chem 165–166: 21–29 10.1016/j.bpc.2012.03.001 22464849

[20] VanWart AT, Eargle J, Luthey-Schulten Z, Amaro RE (2012) Exploring Residue Component Contributions to Dynamical Network Models of Allostery. J Chem Theory Comput 8: 2949–2961 10.1021/ct300377a 23139645 PMC3489502

[21] Tikhonova IG, Selvam B, Ivetac A, Wereszczynski J, McCammon JA (2013) Simulations of Biased Agonists in the β2 Adrenergic Receptor with Accelerated Molecular Dynamics. Biochemistry 52: 5593–603 10.1021/bi400499n 23879802 PMC3763781

[22] Sol A, Fujihashi H, Amoros D, Nussinov R (2006) Residues crucial for maintaining short paths in network communication mediate signaling in proteins. Mol Syst Biol 1–12 10.1038/msb4100063 PMC1681495 16738564

[23] Gasper P, Fuglestad B (2012) Allosteric networks in thrombin distinguish procoagulant vs. anticoagulant activities. PNAS 109: 21216–21222 10.1073/pnas.1218414109 23197839 PMC3535651

[24] Quick M, Winther A-ML, Shi L, Nissen P, Weinstein H, et al (2009) Binding of an octylglucoside detergent molecule in the second substrate (S2) site of LeuT establishes an inhibitor-bound conformation. Proc Natl Acad Sci U S A 106: 5563–5568 10.1073/pnas.0811322106 19307590 PMC2667088

[25] Mondal S, Khelashvili G, Shi L, Weinstein H (2013) The cost of living in the membrane: a case study of hydrophobic mismatch for the multi-segment protein LeuT. Chem Phys Lipids 169: 27–38 10.1016/j.chemphyslip.2013.01.006 23376428 PMC3631462

[26] Bell AJ (2003) The co-information lattice. 4th Int Sympoosium Indep Compon Anal Blind Signal Sep: 921g926.

[27] McGill WJ (1954) Multivariate Information Transmission. Inf Theory, Trans IRE Prof Gr 4: 93–111.

[28] Matsuda H (2000) Physical nature of higher-order mutual information: intrinsic correlations and frustration. Phys Rev E Stat Phys Plasmas Fluids Relat Interdiscip Topics 62: 3096–3102 10.1103/PhysRevE.62.3096 11088803

[29] Sakaguchi M (1967) Interaction information in multivariate probability distributions. Kodai Math Semin Reports 19: 147–155 10.2996/kmj/1138845386

[30] Cover T.M. and Thomas JA (1991) Elements of information theory. New York, NY: John Wiley & Sons.

[31] Watanabe S (1960) Information Theoretical Analysis of Multivariate Correlation. IBM J Res Dev 4: 66–82.

[32] Garner W (1962) Uncertainty and Structure as Psychological Concepts. New York, NY: John Wiley & Sons.

[33] Margolin aa, Wang K, Califano a, Nemenman I (2010) Multivariate dependence and genetic networks inference. IET Syst Biol 4: 428–440 10.1049/iet-syb.2010.0009 21073241

[34] Chae PS, Rasmussen SGF, Rana RR, Gotfryd K, Chandra R, et al (2010) Maltose-neopentyl glycol (MNG) amphipgiles for solubilization, stabilization and crystallization of membrane proteins. Nat Methods 7: 1003–8 10.1038/nMeth.1526 21037590 PMC3063152

[35] Chung KY, Kim TH, Manglik A, Alvares R, Kobilka BK, et al (2012) The role of detergents on conformational exchange of a G protein-coupled receptor. J Biol Chem 1–19 10.1074/jbc.M112.406371 PMC3476297 22893704

[36] Brooks BR, Brooks CL, Mackerell AD, Nilsson L, Petrella RJ, et al (2009) CHARMM: the biomolecular simulation program. J Comput Chem 30: 1545–1614 10.1002/jcc.21287 19444816 PMC2810661

[37] Klauda JB, Venable RM, Freites JA, O'Connor JW, Tobias DJ, et al (2010) Update of the CHARMM all-atom additive force field for lipids: validation on six lipid types. J Phys Chem B 114: 7830–7843 10.1021/jp101759q 20496934 PMC2922408

[38] Phillips JC, Braun R, Wang W, Gumbart J, Tajkhorshid E, et al (2005) Scalable molecular dynamics with NAMD. J Comput Chem 26: 1781–1802 10.1002/jcc.20289 16222654 PMC2486339

[39] Kroemer H, Kittel C (1980) Thermal Physics. 2nd ed. W. H. Freeman Company.

[40] Glykos NM (2006) Software news and updates. Carma: a molecular dynamics analysis program. J Comput Chem 27: 1765–1768 10.1002/jcc.20482 16917862

[41] Lange OF, Grubmüller H (2006) Generalized correlation for biomolecular dynamics. Proteins 62: 1053–1061 10.1002/prot.20784 16355416

[42] Karplus M, Kushick JN (1981) Method for estimating the configurational entropy of macromolecules. Macromolecules 14: 325–332 10.1021/ma50003a019

[43] Andricioaei I, Karplus M (2001) On the calculation of entropy from covariance matrices of the atomic fluctuations. J Chem Phys 115: 6289 10.1063/1.1401821

[44] Piscitelli CL, Gouaux E (2012) Insights into transport mechanism from LeuT engineered to transport tryptophan. EMBO J 31: 228–235 10.1038/emboj.2011.353 21952050 PMC3252571

[45] Claxton DP, Quick M, Shi L, de Carvalho FD, Weinstein H, et al (2010) Ion/substrate-dependent conformational dynamics of a bacterial homolog of neurotransmitter∶sodium symporters. Nat Struct Mol Biol 17: 822–829 10.1038/nsmb.1854 20562855 PMC3245867

[46] Quick M, Shi L, Zehnpfennig B, Weinstein H, Javitch Ja (2012) Experimental conditions can obscure the second high-affinity site in LeuT. Nat Struct Mol Biol 19: 207–211 10.1038/nsmb.2197 22245968 PMC3272158

[47] Khelashvili G, Levine MV, Shi L, Quick M, Javitch Ja, et al (2013) The Membrane Protein LeuT in Micellar Systems: Aggregation Dynamics and Detergent Binding to the S2 Site. J Am Chem Soc 135: 14266–14275 10.1021/ja405984v 23980525 PMC3788620

[48] Wang H, Gouaux E (2012) Substrate binds in the S1 site of the F253A mutant of LeuT, a neurotransmitter sodium symporter homologue. EMBO Rep 1–6 10.1038/embor.2012.110 PMC3432802 22836580

[49] Moradi M, Tajkhorshid E (2013) Mechanistic picture for conformational transition of a membrane transporter at atomic resolution. Proc Natl Acad Sci U S A 110: 18916–18921 10.1073/pnas.1313202110 24191018 PMC3839739

[50] Celik L, Schiøtt B, Tajkhorshid E (2008) Substrate binding and formation of an occluded state in the leucine transporter. Biophys J 94: 1600–1612 10.1529/biophysj.107.117580 18024499 PMC2242742

[51] Koldsø H, Autzen HE, Grouleff J, Schiøtt B (2013) Ligand induced conformational changes of the human serotonin transporter revealed by molecular dynamics simulations. PLoS One 8: e63635 10.1371/journal.pone.0063635 23776432 PMC3680404

[52] Piscitelli CL, Krishnamurthy H, Gouaux E (2010) Neurotransmitter/sodium symporter orthologue LeuT has a single high-affinity substrate site. Nature 468: 1129–1132 10.1038/nature09581 21179170 PMC3079577

[53] Krishnamurthy H, Gouaux E (2012) X-ray structures of LeuT in substrate-free outward-open and apo inward-open states. Nature 481: 469–474 10.1038/nature10737 22230955 PMC3306218

[54] Wang H, Goehring A, Wankg KH, Penmatsa A, Ressler R, et al (2013) Structural basis for action by diverse antidepressants on biogenic amine transports. Nature 503: 141–5 Doi: 10.1038/nature12648. 24121440 10.1038/nature12648 PMC3904662

[55] Singh SK, Piscitelli CL, Yamashita A, Gouaux E (2008) A competitive inhibitor traps LeuT in an open-to-out conformation. Science (80- ) 322: 1655–1661 10.1126/science.1166777 PMC2832577 19074341

[56] Beuming T, Shi L, Javitch JA, Weinstein H (2006) A Comprehensive Structure-Based Alignment of Prokaryotic and Eukaryotic Neurotransmitter/Na+ Symporters ( NSS ) Aids in the Use of the LeuT Structure to Probe NSS Structure and Function. 70: 1630–1642 10.1124/mol.106.026120.targets 16880288

