# Supplementary material for: NbIT - A New Information Theory-Based Analysis of Allosteric Mechanisms Reveals Residues that Underlie Function in the Leucine Transporter LeuT
Source: PLoS Comput Biol. 2014 May 1;10(5):e1003603. doi: 10.1371/journal.pcbi.1003603 (PMC4006702; doi:10.1371/journal.pcbi.1003603)
Supplement: File S1 — Supporting Methods (MD simulations; Moving block bootstrapping of MD simulations; Contributions for specific information measures; Identifying high information residues); Supporting Discussion (Efficient information transmission; Normalizing mutual information; Negative co-information ; Analysis of the K1,4 network; Control study); Tables S1–S4; Figures S1–S11; Supporting References. (PDF) [file pcbi.1003603.s001.pdf]

SUPPORTING INFORMATION

NbIT - a new information theory-based analysis of allosteric mechanisms reveals residues that underlie function in the leucine transporter LeuT.

Michael V. LeVine and Harel Weinstein

SUPPORTING METHODS

MD Simulations

<u>LeuT$_{POPE/POPG}$</u>: In the LeuT$_{POPE/POPG}$ simulation the occluded LeuT structure [1] (PDB ID 3GJD) bound to the two sodium ions and leucine, but with the octyl-glucoside (OG) detergent molecule removed, is embedded in a bilayer membrane model composed 534 POPE and 160 POPG lipids, fully solvated in TiP3P water and 0.15 mM NaCl. The details of the simulation and results are given in [2].

<u>LeuT$_{MNG-3}$</u>: The simulated system was generated from the LeuT$_{POPE/POPG}$ system by removing the equilibrated protein from the membrane and creating a micelle of 120 MNG-3 molecules around it using the molecular packing algorithm *packmol* [3]. The MNG-3 molecule was created using Maestro 9.3 [4] and parameterized using MATCH [5] to assemble parameters from previous parameterized sugars and lipids. The system was solvated and ionized in 0.15 mM NaCl.

<u>LeuT$_{APO}$</u>: Dr. Lei Shi provided the MD trajectory from a substrate-free simulation constructed from the original occluded structure (PDB ID 2A65) embedded in a membrane model composed of POPC lipids.

Moving block Bootstrapping of MD Simulations

In our analysis, the estimated entropy is a function of the number of observations available because it is derived from the covariance matrix which is sensitive to number of observations [6]. The average root mean square fluctuations grow with time [7] and so does the number of observations. Thus, comparing information calculations between trajectories requires an equivalent number of observations. Another consideration is that MD is a dynamical process, and hence there is high correlation between consecutive frames in a simulation that can lead to errors in the estimate of statistical quantities. To address both considerations, we used moving block bootstrapping in order to down-sample our simulations to the equivalent number of frames, and to achieve a better estimate of the error in our calculations due to autocorrelation. As described in [8], we used moving block bootstrapping with block sizes of 500, 625,

1000, 1250, and 2500 to resample 50 new trajectories for each block size, and then evaluated for each calculation the maximum standard error on the mean for all block sizes. It should be noted that moving block bootstrapping was used after removing the open state frames, and thus some frames within blocks are not consecutive in the original simulation. For the calculations presented here, $LeuT_{POPE/POPG}$ and $LeuT_{MNG-3}$ were down-sampled to 5000 frames from 5447 and 5657 frames, respectively.

Calculating Contributions for Specific Information Measures

To calculate the contribution of a specific residue, Z, to the *mutual information* between a set of residues, X_1, and another set of residues, X_2, we calculate the *mutual information* between the set difference of X_1 and Z and the set difference of X_2 and Z, conditional on Z, and normalize it to the mutual information between X_1 and X_2:

$$Contribution_{I_2}(x) = \frac{I_2(X_1, X_2) - I_2(X_1 - Z, X_2 - Z)|Z}{I_2(X_1, X_2)} * 100\% \quad \text{(S.1)}$$

To calculate the contribution of a specific residue, Z, to the *total correlation* between a set of residues, X_1, we calculate the *total correlation* of the set difference of X_1 and Z, conditional on Z, and normalize it to the *total correlation* of X_1.

$$Contribution_{TC}(x) = \frac{TC(X_1) - TC(X_1 - Z)|Z}{TC(X_1)} * 100\% \quad \text{(S.2)}$$

To calculate the contribution of a specific residue, Z, to the *coordination information* between a set of residues X_1 and a set of residues X_2, we calculate the *coordination information* between the set difference of X_1 and Z and the set difference of X_2 and Z, conditional on Z, and normalized it to the *coordination information* between X_1 and X_2. This form generalizes the equation to describe the contribution of a residue acting as a coordinator or being coordinated.

$$Contribution_{CI}(x) = \frac{CI(X_1, X_2) - CI(X_1 - Z, X_2 - Z)|Z}{CI(X_1, X_2)} * 100\% \quad \text{(S.3)}$$

Identifying High Co-information Residues

To identify the residues with high *co-information*, we take advantage of the relationship between the *co-information* and the *co-information rank*. We find that when plotting *co-information* against the *co-information rank* for an arbitrary pair of sites, the midsection contains a large linear region, surrounded with a large co-information extreme on the left and a small co-information extreme on the right. To identify a cut-off for the high *co-information* extreme region, we calculate a linear fit to the middle 200 residues and calculate the root mean squared residual (RMSR) for the fit. We then project the fit across all

residues, and define the residues with high *co-information* as those that have a residual of greater than 1 RMSR. An example distribution and the corresponding fit and cut-off are shown in Fig. S4. This method has been used previously [9].

Efficient Information Transmission

We illustrate with the use of a simple two-dimensional three-body system (Fig. S1), that if the axes of covariance are not aligned, the consecutive pairwise correlations may be high, but the system cannot transmit information. Thus, if A and B in Fig. S1 co-vary on the blue axis, information about the position of A on the blue axis is present in the position of B on the blue axis, and if B co-varies with C on the red axis, information about the position of B on the red axis is present in the position of C on the red axis. When A and C co-vary with B on different axes, no information about the position of B on the blue axis is present in the position of C on the red axis, and thus no information about the position of A on the blue axis is present in the position of C on the red axis, i.e., there is no allosteric information transmission (Fig. S1, left).

However, when the blue and red axes are aligned, A, B and C all co-vary on the same axis and the 3-body correlation leads to information about the position of A on the blue axis being present in the position of C on the red axis (Fig. S1, right). This model illustrates a weakness in the use of network theoretical methods that do not maximize the higher n-body correlations: while shortest path analysis maximizes the pairwise correlations, one would expect that many pathways found using such network theoretical methods may not actually be efficient information channels.

Normalizing Mutual Information

Several normalizations of mutual information have been proposed in the past [10], but they all rely on entropy being positive. The analytical approximation of entropy, *differential entropy*, is not strictly positive, and for the high dimensional distributions studied here, it is almost always negative. Because of the negative entropies encountered here, normalization by standard methods is not possible. One additional method has been proposed, the generalized correlation coefficient [11], which relies on the relationship between mutual information and Pearson correlation coefficient for univariate normal distributions. However, the generalization to higher dimensions it provides is not rigorous, and the measure is unreliable at high dimensions when there is significant *total correlation* within each distribution. For illustration, imagine two unimodal normal distributions with 1 bit of entropy each that are coupled with mutual information of 0.9 bits. If each distribution is extended to two dimensions, where the second dimension has 1 bit of entropy and the two dimensions are nearly perfectly coupled with

mutual information of 0.9 bits, the entropy of each 2-dimensional distribution is now 1.1 bits, and the mutual information between the two 2-dimensional distributions will be at most 1 bit. As the mutual information between the first and second dimension approaches the entropy of the second dimension, the increase in mutual information between the distributions when each distribution is made 2-dimensional converges to 0. Thus, although the system is beginning to act as two coupled rigid bodies, as the dimension of the distributions grows past 2, the generalized correlation coefficient between them shrinks because the gain in mutual information between them is minimal. This demonstrates that the generalized correlation coefficient is not appropriate for high dimensional distributions where there is significant total correlation between dimensions. For this reason we do not compare mutual information between different *frc* pairs. However, the other co-information metrics described here can be normalized, as described in Methods, and we discuss and compare them using their normalized values.

Negative Co-information

If variables X_1 and X_2 are positively correlated by direct interaction, but X_2 is positively correlated to X_3 while X_1 is negatively correlated to X_3 (both by direct interaction), the information shared by X_1 and X_2 is diminished due to their interaction with X_3 for certain parameters (for example, when the correlation between X_1 and X_2 is 0.1, the correlation between X_1 and X_3 is -0.7, and the correlation between X_2 and X_3 is 0.7). While this can occur in allosteric biomolecular systems, we have found it to be rare. The phenomenon also occurs in coordination information, where negative coordination information indicates that the variable de-coordinates the set of residues via the mechanism described for co-information; however, we do not observe this phenomenon in our analysis.

Analysis of the $K_{1,4}$ Network

MD simulations can produce trajectories containing from thousands to hundreds of thousands of observations of the system, and while this offers substantially more observations than are required for most statistical analyses, we sought to validate the ability of NbIT analysis to identify channels from *co-information* and *coordination information* alone, without explicitly considering the underlying topology of the network.

In order to illustrate the power of NbIT analysis, we designed symmetric $K_{1,4}$ networks of coupled univariate normal distributions (see Fig. S5, top left). Each of the three illustrative systems - weak, moderate, and strong - is defined by a covariance matrix with the diagonal elements (the variances) equal to 1. In the weak system, the covariance between directly coupled distributions, r_{direct}, is 0.25 and the covariance between indirectly coupled distributions, $r_{indirect}$, is 0.0625. In the moderate system, r_{direct} = 0.5 and $r_{indirect}$ = 0.25, and in the strong one, r_{direct} = 0.75 and $r_{indirect}$ = 0.2625. For each system, inverting the

covariance matrix produces 0 in all the elements corresponding to interactions between indirectly coupled distributions, which indicates that they are conditionally independent as intended.

To illustrate the ability of the measures used in NbIT analysis to define channels and coordinators, we sampled the complete multivariate distribution with numbers of observations ranging from 200 to 10000 times (in multiples of 200), each with ten realizations, using the **mvrnorm** function within the R package **MASS**. The tests were: 1) how well one could differentiate node 1, which is the true channel between nodes 2 and 3, from node 4, which is a false channel, by using *co-information*; and 2) how well one could differentiate node 1 - the true coordination channel for the coordination of nodes 2 and 3 by node 5 - from node 4, a false coordination channel, using *mutual coordination information.* The results are summarized in Fig. S5 showing that even for the weak system, where the true indirect correlations are lower than would traditionally be considered for investigation, both *co-information* and *coordination information* can determine the true channels from the false channels if one has over 8000 observations of the systems. For the moderate system, channels can be identified with fewer than 2000 observations. These results indicate that channels can be identified by the criteria of *co-information* or *coordination information* using the number of observations typically available to MD simulations.

Control study: Evaluate the coordination of intracellular domains lacking specific functional roles in the allosteric process

Because there is no standard to assess the amount of *normalized coordination information* (NCI) that is required for biological significance, we calculated for comparison purposes the normalized coordination information between the substrate sites S1 and S2 and several structures in the intracellular domain that were chosen arbitrarily, without relation to any kind of functional information. Five such controls were performed, involving the following residue groups depicted in Fig S7: residues 506-511 - the helical region at the far intracellular end of TM12, which is distant from the center of the INI (control 1); residues 65-70 - a helical region of TM2 proximal to the INI (control 2); residues 77 – 96 - a helix-helix packing interface proximal to the INI (control 3); residues 269-275 and 437-445 - a loop-loop packing interface proximal to the INI (control 4); and residues 11-22 of TM1a, which is a TM domain proximal to the INI and S1 (control 5). In addition, for the packing interfaces, each interface was broken down into 3 control sets: each component of the packing interface individually, the entire set of residues, and the residues directly packing. We found in all cases that the S1 and S2 *frc*-s displayed significantly less *normalized coordination information* with any of the control residue groups, than they did with the INI, demonstrating the significance of the results reported for the functionally relevant *frc*-s. Results are summarized in Table S1.

**A. LeuT$_{POPE/POPG}$**						
**S1**	**1. 506-511**	**2. 65-70**	**3A. 77-84**	**3B. 87-96**	**3C. 77-96**	**3D. 78, 81-82, 85-87, 90, 91, 94**
	5.4% (0.2%)	5.0% (0.2%)	4.0% (0.1%)	4.8% (0.1%)	3.3% (0.2%)	4.0% (0.2%)
	**3E. 78, 81-82, 90, 91, 94**	**4A. 437-445**	**4B. 269-274**	**4C. 437-445, 269-274**	**4D. 438-440, 272-274**	**5. 11-22**
	5.9% (0.1%)	2.6% (0.1%)	4.7% (0.2%)	3.0% (0.2%)	4.2% (0.1%)	10.7% (0.2%)
**S2**	**1. 506-511**	**2. 65-70**	**3A. 77-84**	**3B. 87-96**	**3C. 77-96**	**3D. 78, 81-82, 85-87, 90, 91, 94**
	10.7% (0.3%)	4.4% (01%)	7.1% (0.2%)	6.8% (0.1%)	5.4% (0.3%)	7.0% (0.2%)
	**3E. 78, 81-82, 90, 91, 94**	**4A. 437-445**	**4B. 269-274**	**4C. 437-445, 269-274**	**4D. 438-440, 272-274**	**5. 11-22**
	9.3% (0.2%)	3.7% (0.1%)	4.8% (0.2%)	3.6% (0.2%)	4.6% (0.2%)	6.2% (0.2%)
**B. LeuT$_{MNG-3}$**						
**S1**	**1. 506-511**	**2. 65-70**	**3A. 77-84**	**3B. 87-96**	**3C. 77-96**	**3D. 78, 81-82, 85-87, 90, 91, 94**
	5.9% (0.2%)	6.5% (0.3%)	6.3% (0.2%)	7.9% (0.2%)	5.9% (0.3%)	8.3% (0.2%)
	**3E. 78, 81-82, 90, 91, 94**	**4A. 437-445**	**4B. 269-274**	**4C. 437-445, 269-274**	**4D. 438-440, 272-274**	**5. 11-22**
	10.2% (0.3%)	7.3% (0.3%)	7.7% (0.2%)	7.2% (0.3%)	8.8% (0.3%)	13.9% (0.3%)
**S2**	**1. 506-511**	**2. 65-70**	**3A. 77-84**	**3B. 87-96**	**3C. 77-96**	**3D. 78, 81-82, 85-87, 90, 91, 94**
	7.6% (0.2%)	6.1% (0.2%)	6.8% (0.2%)	8.2% (0.2%)	6.3% (0.3%)	9.5% (0.2%)
	**3E. 78, 81-82, 90, 91, 94**	**4A. 437-445**	**4B. 269-274**	**4C. 437-445, 269-274**	**4D. 438-440, 272-274**	**5. 11-22**
	10.9% (0.2%)	7.7% (0.3%)	8.5% (0.2%)	7.8% (0.3%)	9.3% (0.3%)	8.8% (0.2%)

Table S1. Coordination of control regions by S1 and S2 in LeuT$_{POPE/POPG}$ and LeuT$_{MNG-3}$. The coordination of each control region by S1 and S2 are presented with the standard error of the mean in parenthesis, estimated using moving block bootstrapping with 50 realizations.

$LeuT_{POPE/POPG}$ Clustering						
	S1	S2	Na1	Na2	INI	ALL
Total SS	168295	104439	89173.11	17265.54	1032241	2890429
Between SS/Total SS	0.30	0.21	0.71	0.35	0.84	0.54
Overlap with ALL	85.3%	42.9%	98.3%	92.3%	96.8%	100.0%
$LeuT_{MNG-3}$ Clustering						
	S1	S2	Na1	Na2	INI	ALL
Total SS	95297.29	110957.7	51345.57	21880.25	446284.2	1472776
Between SS/Total SS	0.17	0.35	0.46	0.47	0.78	0.36
Overlap with ALL	54.4%	57.2%	99.8%	49.4%	99.6%	100.0%

Table S2. Clustering by frc-s in $LeuT_{POPE/POPG}$ and $LeuT_{MNG-3}$. Clustering of the simulations using the pairwise distances between members in a given frc was performed with 100 iterations of k-means clustering. For each clustering criterion, we calculate the total sum of square distances between frames (Total SS), the fraction of the total SS that is contributed by distance between conformations in different clusters (Between SS/Total SS), and the overlap (as a percentage) calculated using Eq. 1.

	S1	S2	Na1	Na2	Na1 Na2	Na1 Na2 S1	Na1 Na2 S1 S2	INI
S1	***31.7*** **(0.2)**	24.1% (0.5%)	29.3% (0.4%)	17.1% (0.3%)	32.9% (0.4%)	X	X	11.9% (0.4%)
S2	15.5% (0.3%)	***32.5*** **(0.3)**	9.6% (0.2%)	4.8% (0.2%)	9.7% (0.3%)	16.2% (0.4%)	X	6.7% (0.2%)
Na1	58.2% (0.8%)	49.2% (0.6%)	***9.7*** **(0.1)**	39.8% (0.6%)	X	X	X	22.0% (1.0%)
Na2	36.1% (0.2%)	14.5% (0.2%)	32.8% (0.2%)	***12.3*** **(0.0)**	X	X	X	8.5% (0.3%)
Na1 Na2	30.7% (0.3%)	24.6% (0.4%)	X	X	***30.5*** **(0.1)**	X	X	12.5% (0.5%)
Na1 Na2 S1	X	16.6% (0.4%)	X	X	X	***68.7*** **(0.1)**	X	9.9% (0.5%)
Na1 Na2 S1 S2	X	X	X	X	X	X	***133.6*** **(1.5)**	6.7% (0.3%)
INI	17.3% (0.5%)	16.4% (0.6%)	13.8% (0.6%)	9.0% (0.4%)	15.2% (0.6%)	20.2% (0.6%)	22.7% (0.9%)	***13.3*** **(0.1)**

Table S3. Normalized Coordination Information between sites in LeuT$_{MNG-3}$. For each pair of *frc*-s, the normalized coordination information is presented, with residues on the top (columns) acting as the coordinator and residues on the left (rows) being coordinated. On the diagonal, total correlation of the site is shown in bold. The standard error of the mean calculated using moving block bootstrapping is shown in parenthesis.

S1	Leu	L25	G26	V104	Y108	F253
	**40.8% (0.3%)**	35.9% (0.2%)	31.4% (0.2%)	13.9% (0.2%)	24.8% (0.3%)	**39.8% (0.2%)**
	T254	S256	F259	S355	I359	Na1
	**43.2% (0.2%)**	27.6% (0.2%)	17.0% (0.1%)	12.5% (0.1%)	13.9% (0.1%)	22.8% (0.3%)
S2	L29	R30	Y107	I111	W114	F253
	26.0% (0.1%)	**28.3% (0.1%)**	20.9% (0.1%)	20.1% (0.2%)	18.9% (0.1%)	10.7% (0.1%)
	A319	F320	F324	L400	D404	
	**32.2% (0.2%)**	**39.0% (0.2%)**	22.7% (0.2%)	14.4% (0.1%)	16.1% (0.1%)	
Na1	Na1	A22	N27	T254	N286	Leu
	**63.2% (0.4%)**	45.3% (0.4%)	51.1% (0.2%)	**63.8% (0.4%)**	31.2% (0.4%)	**61.4% (0.4%)**
Na2	Na2	G20	V23	A351	T354	S355
	**48.1% (0.1%)**	38.5% (0.1%)	38.0% (0.1%)	35.1% (0.1%)	**68.3% (0.1%)**	**64.8% (0.1%)**
INI	R5	I187	S267	Y268	Q361	D369
	**43.5% (0.4%)**	24.8% (0.5%)	**63.8% (0.3%)**	**66.1% (0.2%)**	16.1% (0.3%)	24.6% (0.4%)

Table S4. The contribution of specific residues to the total correlation of their sites in LeuT$_{MNG-3}$. For each site, the contribution of each residue to the total correlation in LeuT$_{MNG-3}$ is presented. The top 3 contributors in each site are shown in bold.

A. Contribution to the Coordination of the INI by S1						
S1	Leu	L25	G26	V104	Y108	F253
	27.8% (1.0%)	25.6% (0.6%)	21.2% (0.7%)	23.7% (0.8%)	23.3% (0.9%)	25.3% (1.5%)
	T254	S256	F259	S355	I359	Na1
	**38.6% (1.6%)**	27.2% (1.1%)	**52.4% (1.1%)**	17.6% (0.6%)	**37.8% (0.7%)**	28.3% (1.0%)
INI	R5	I187	S267	Y268	Q361	D369
	**73.0% (1.1%)**	**53.7% (1.4%)**	**60.2% (0.9%)**	**53.9% (0.6%)**	34.6% (0.9%)	48.4% (0.9%)
B. Contribution to the Coordination of the INI by S2						
S2	L29	R30	Y107	I111	W114	F253
	**31.7% (1.5%)**	**37.3% (1.6%)**	26.1% (2.6%)	**32.2% (2.3%)**	13.8% (1.0%)	26.8% (1.1%)
	A319	F320	F324	L400	D404	
	20.1% (1.2%)	19.3% (1.6%)	**30.3% (1.4%)**	20.3% (0.8%)	14.4% (0.9%)	
INI	R5	I187	S267	Y268	Q361	D369
	**80.3% (1.0%)**	**59.0% (1.6%)**	**55.9% (1.6%)**	**54.9% (0.9%)**	22.5% (0.9%)	**53.0% (1.2%)**

Table S5. Specific residues highly contribute to coordination of the INI by S1 in LeuT$_{MNG-3}$. A. The contribution of specific residues in S1 (top) and the INI (bottom) to the coordination of the INI by S1 The top 3 in each site are bold; I187 cannot be statistically distinguished from Y268 (p = 0.90, Welsh's t-test). B. The contribution of specific residues in S2 (top) and the INI (bottom) to the coordination of the INI by S2.The top 3 contributors in each site are bold; Y268 and D359 cannot be statistically distinguished S267 (p = 0.64 and 0.20, respectively, Welsh's t-test).

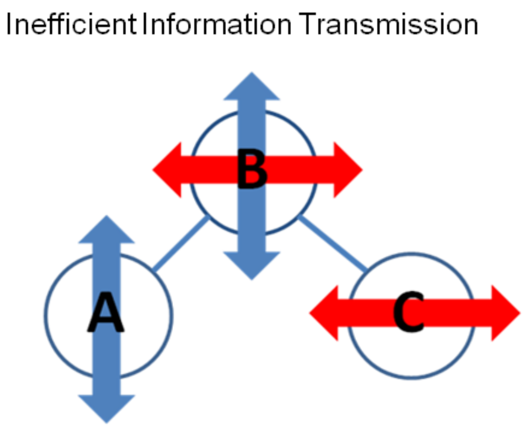

Figure S1. Efficient information transmission by a 3-body system. Information transmission through a 3-body system (solid blue lines represent direct interactions), moving in 2D, is inefficient if the axes of covariance of each pair (thick arrow) is not aligned (left). Information transmission it is efficient if the axes of covariance are aligned (right); the dotted blue lines represent indirect allosteric interaction as a result of information sharing.

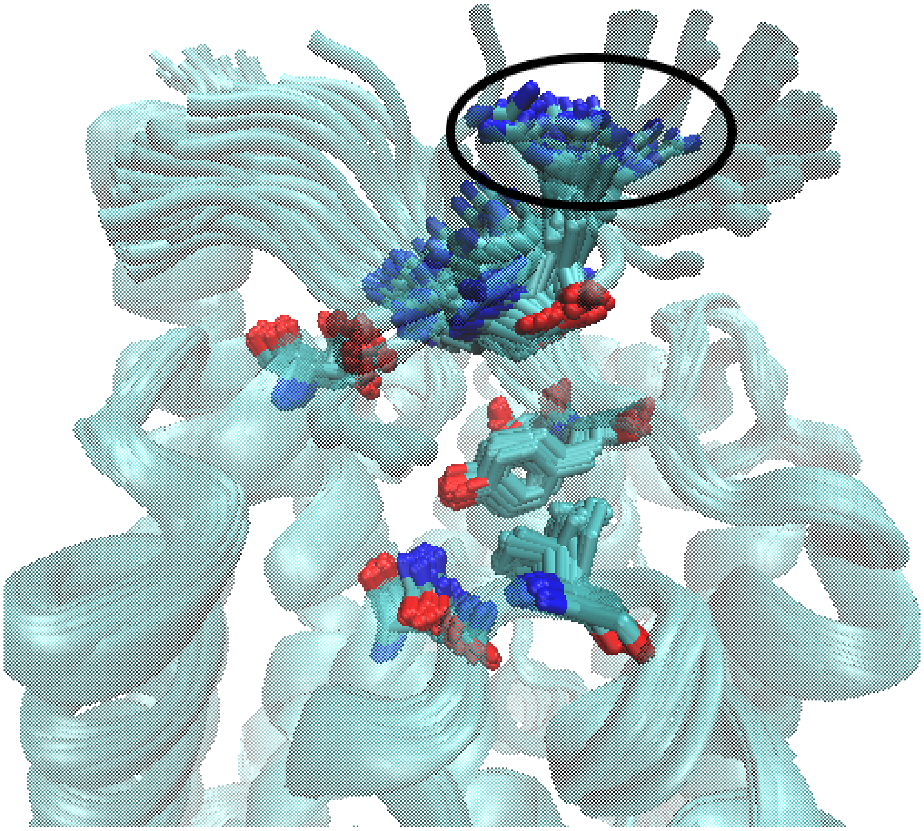

Figure S2. Conformations of the INI. Representative conformations of $LeuT_{POPE/POPG}$. Residues from the INI are shown in licorice representation and the rest of the protein is shown in cyan cartoon representation. The conformations of R5 after transition from the crystallographically observed conformation are outlined.

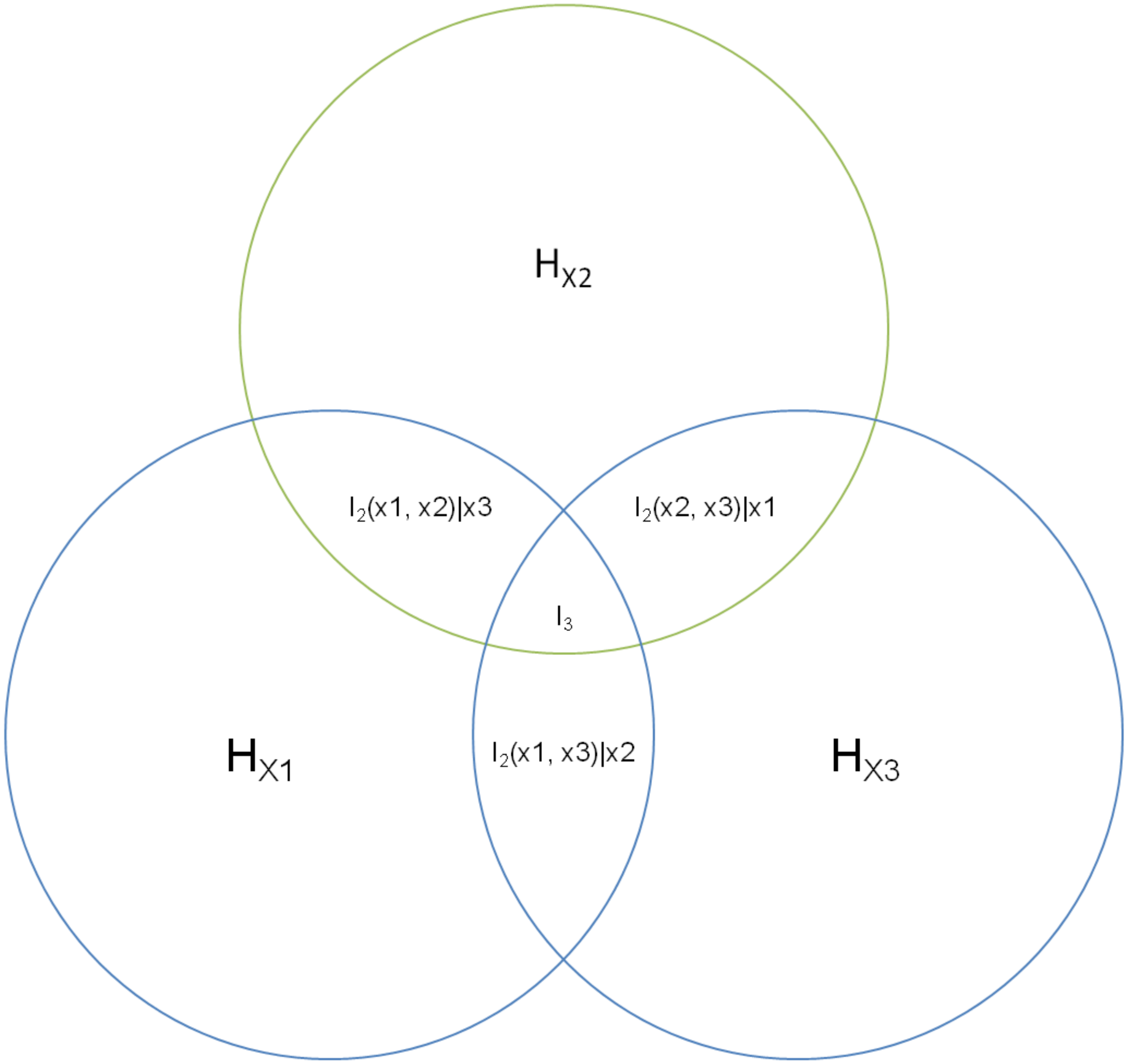

Figure S3. The 3-body information Venn diagram. In a 3-body system, the co-information between three variables is the 3-way intersect, denoted as $I_3(X_1,X_2,X_3)$. Blue circles denote the transmitter and receiver, whereas the green circle denotes the channel.

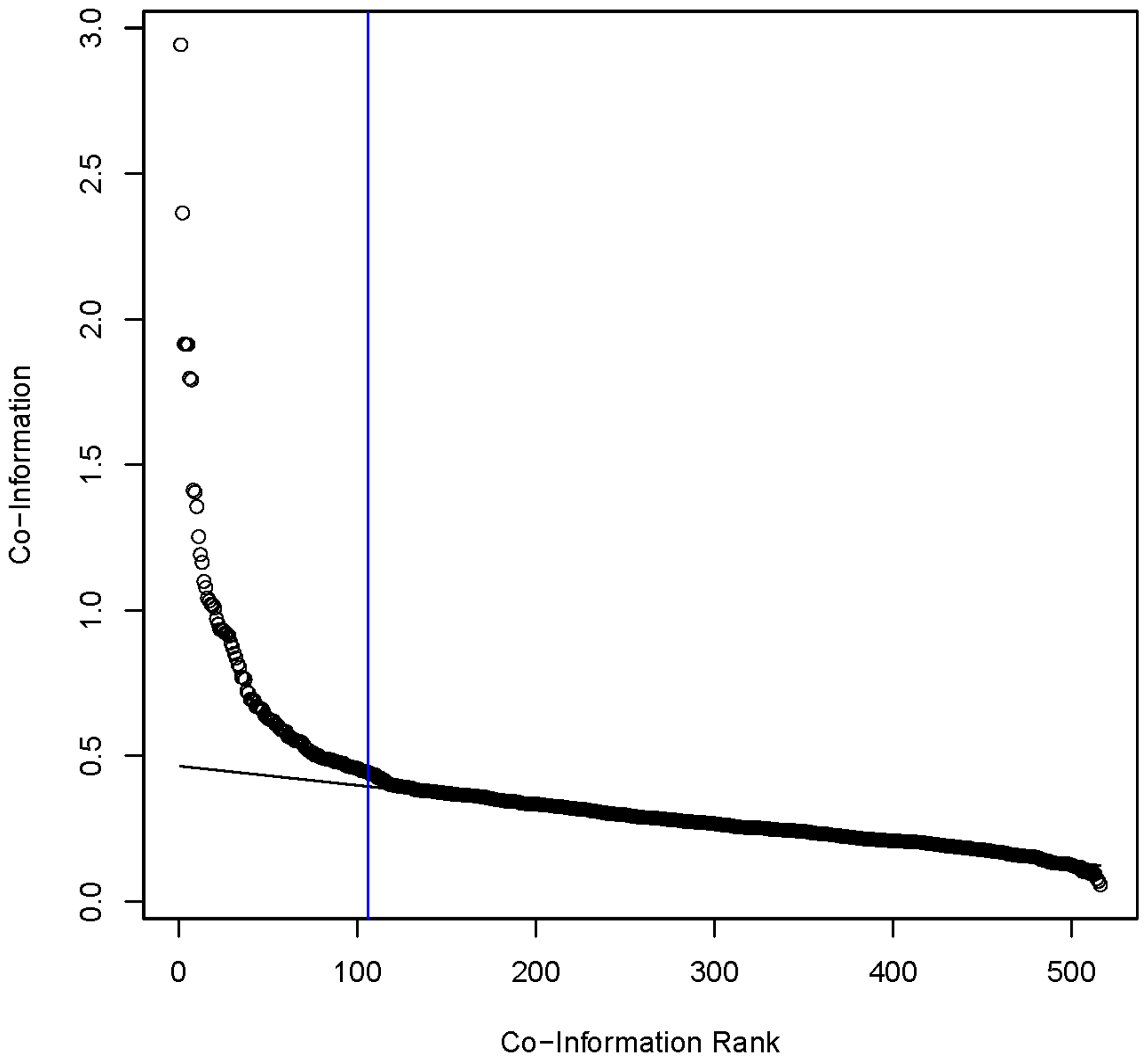

Figure S4. The typical co-information plot. The co-information for a given residue (the channel) with the INI (receiver) and S1 (transmitter) is plotted against the co-information rank of that residue (black circles). The black line is the linear fit to the middle 200 residues and the blue line is the cut-off for high co-information residues.

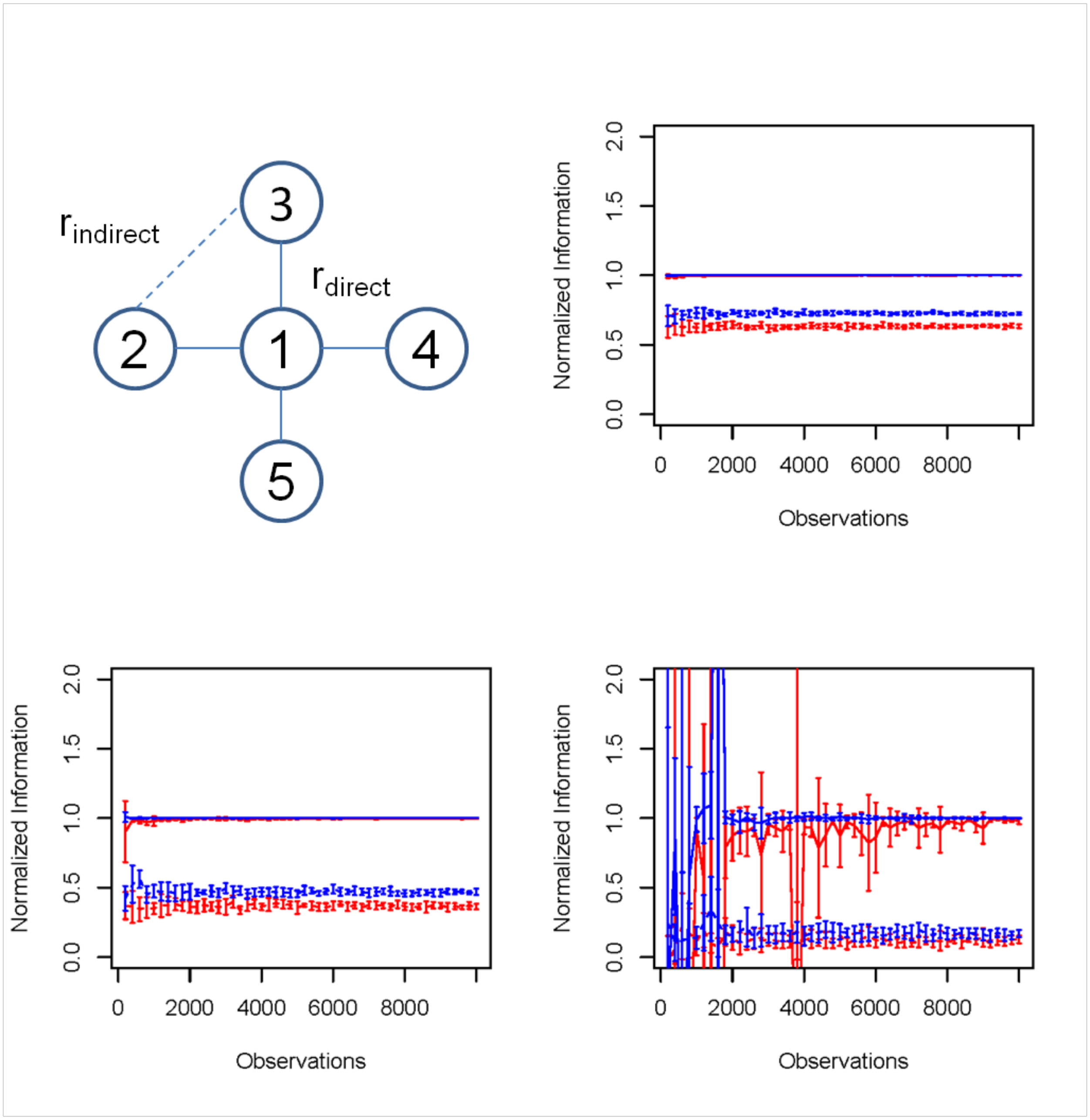

Figure S5. Co-information and Mutual Coordination Information can identify channels in $K_{1,4}$. Top left: The $K_{1,4}$ network that serves to illustrate the ability of these measures to discriminate between true and false channels. Each circle is a node and the connecting lines are the edges. Edges represent direct interactions with covariance r_{direct}, and all nodes that are not connected by an edge display indirect interactions with covariance $r_{indirect}$. Top right: Figure shows the results from separate calculations with different numbers of observations, of normalized co-information (red) and normalized mutual coordination information (blue) in the strong $K_{1,4}$ network. True channels are shown in solid lines and

false channels are shown in dashed lines, and bars represent the standard deviation of 10 realizations. Bottom left: Same as top right, for the moderate $K_{1,4}$ network. Bottom right: Same as left panel, for the weak $K_{1,4}$ network.

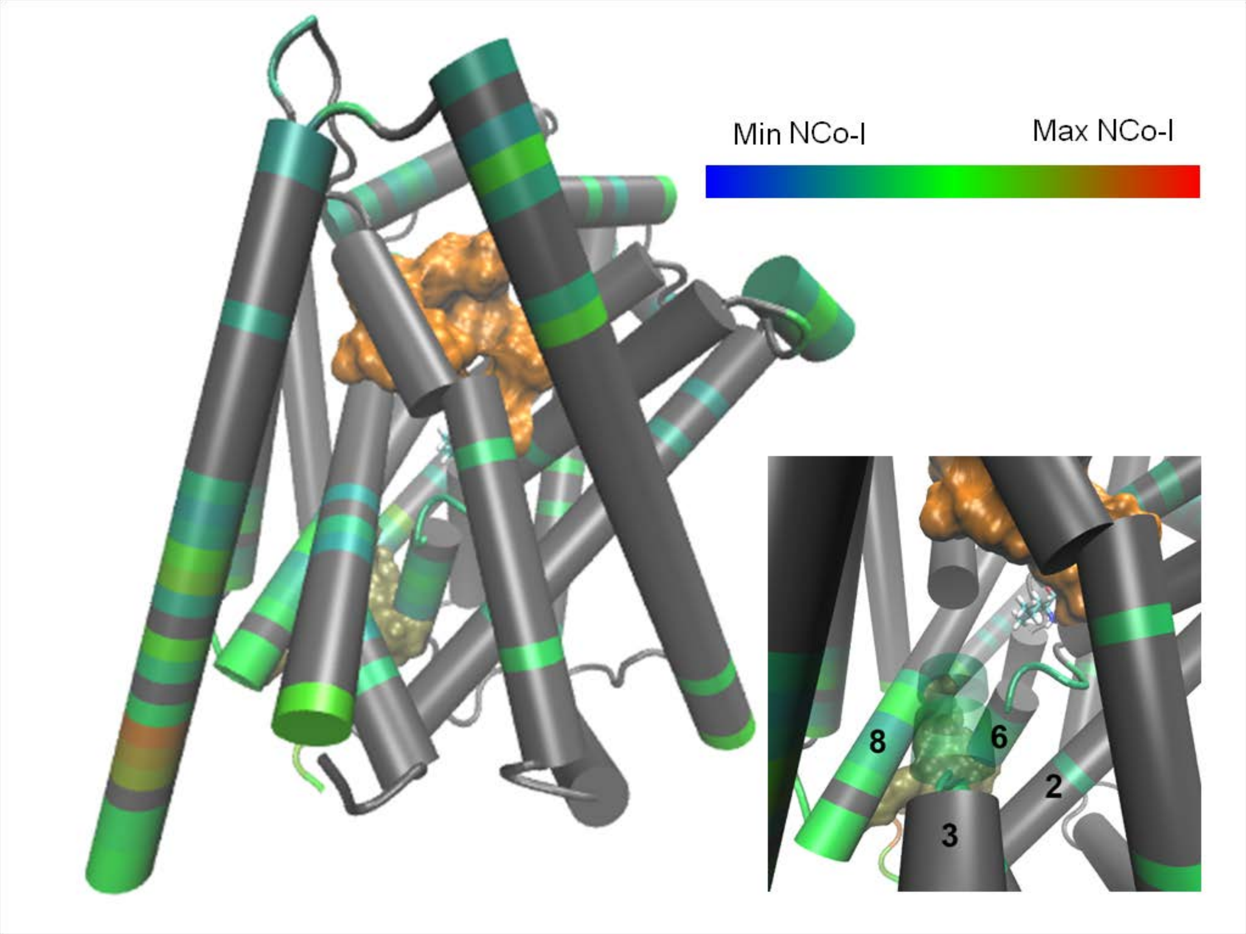

Figure S6. TMs 6b, and 8 form a co-information channel between S2 and the INI. Main: Residues found to have high *co-information* with S1 and the INI are colored by their calculated *normalized co-information* (NCo-I) values using the scale at the top right, where the Min and Max NCo-I refer to the minimum and maximum values among all possible residues. All other residues are represented in grey. Bottom right: A close up of the TM6b and TM8 interface.

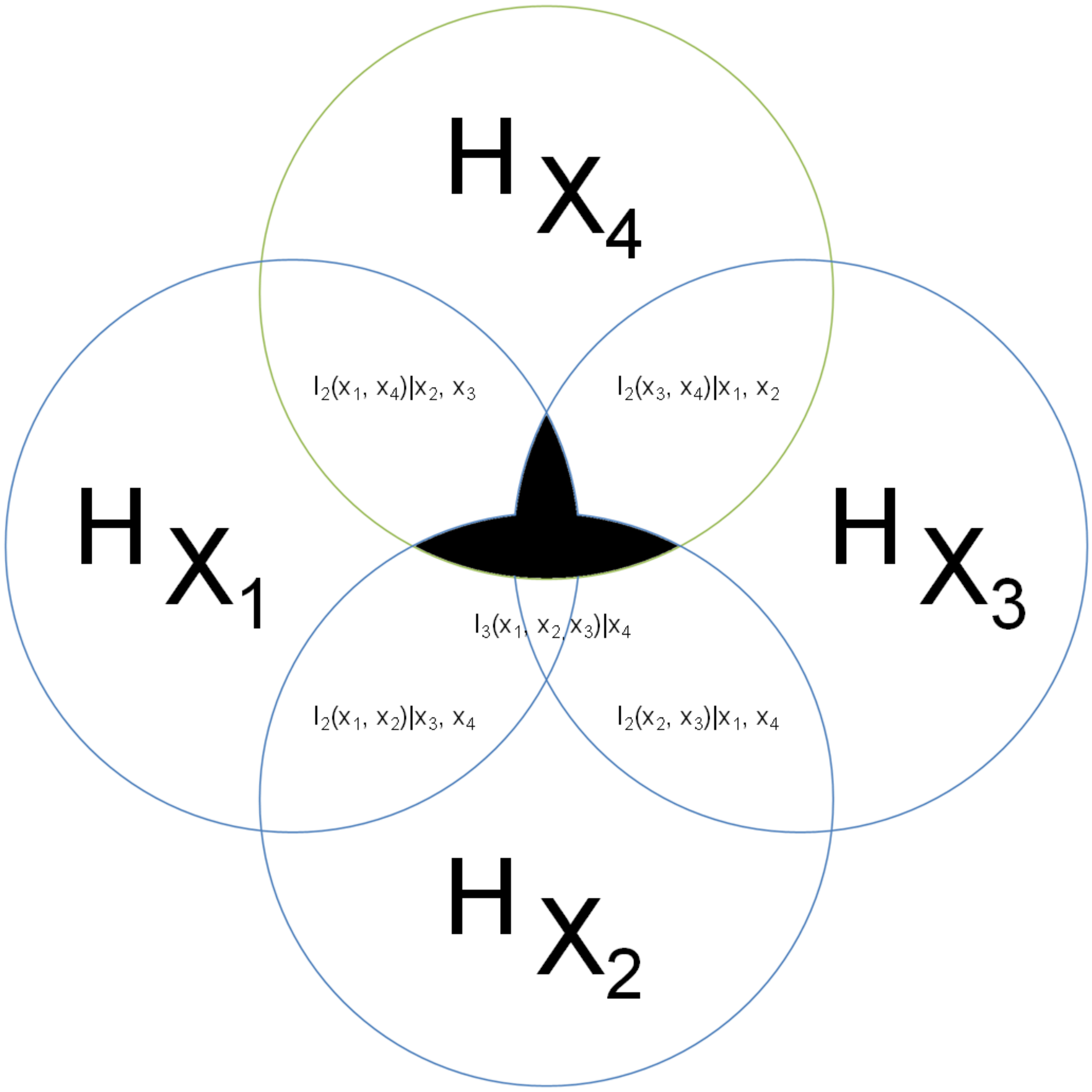

Figure S7. The Venn diagram representation of Coordination Information. Coordination information is the intersect of the entropy of the coordinator (green circle) with the union of all intersections within the set being coordinated (blue circles).

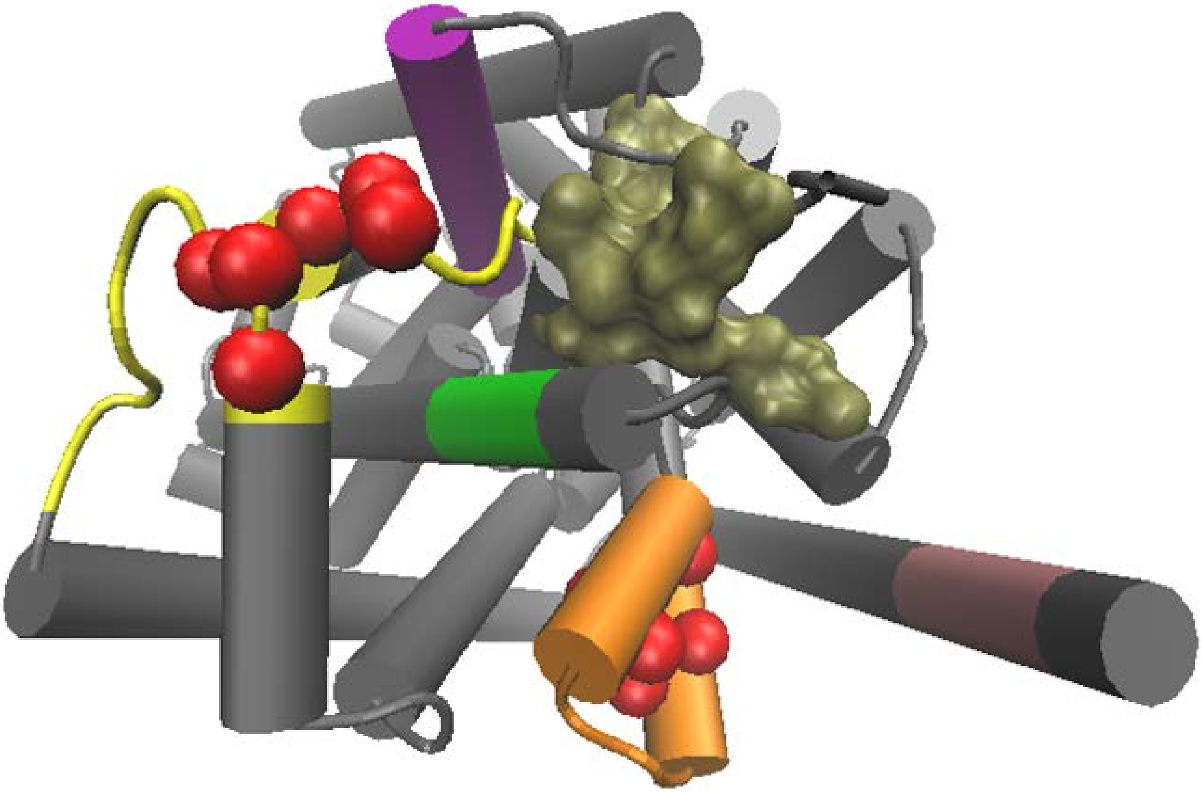

Figure S8. Intracellular regions used in the control calculations. The INI-frc is in gold surface representation, control-1 is in pink, control-2 in green, control-3 is orange (the interface between the two helices is indicated in red VDW), control-4 is yellow (the interface between the two loops is indicated in red VDW), control-5 is in purple.

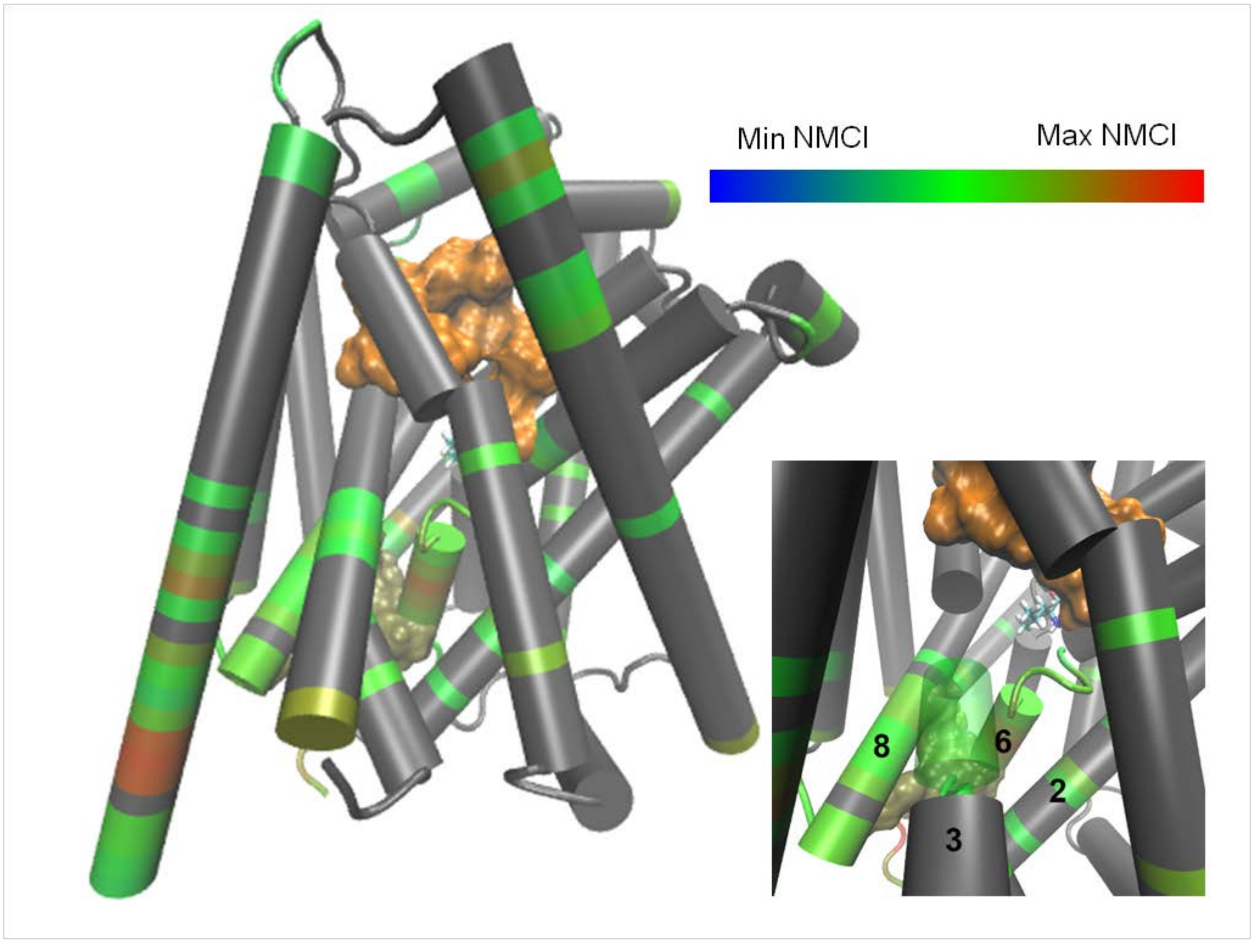

Figure S9. TMs 2, 6b, and 8 form a coordination channel between S2 and the INI in LeuT$_{POPE/POPG}$. Main: Residues found to have high *mutual coordination information* with S2 and the INI are colored by their calculated *normalized mutual coordination information* (NCMI) values using the scale at the top right, where the Min and Max NCMI refer to the minimum and maximum values among all possible residues. All other residues are represented in grey. Bottom right: A close-up of the TM2, TM6b, and TM8 interface.

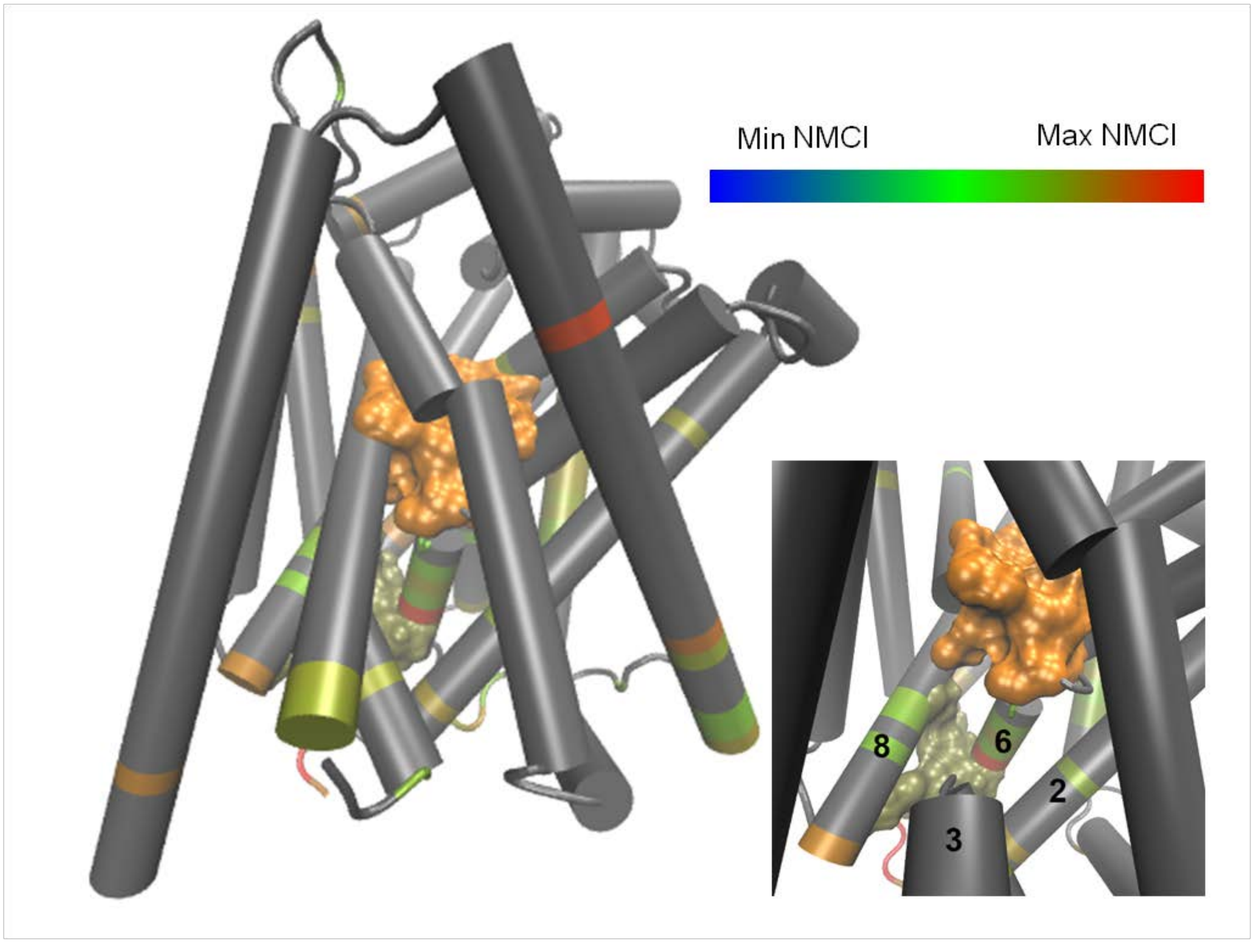

Figure S10. TM 6b forms a coordination channel between S1 and the INI in $LeuT_{MNG-3}$. Main: Residues found to have high *mutual coordination information* with S1 and the INI are colored by their *normalized mutual coordination information* (NCMI) values using the scale at the top right, where the Min and Max NCMI refer to the minimum and maximum values among all possible residues. All other residues are represented in grey. Bottom right: A close-up of TM6b.

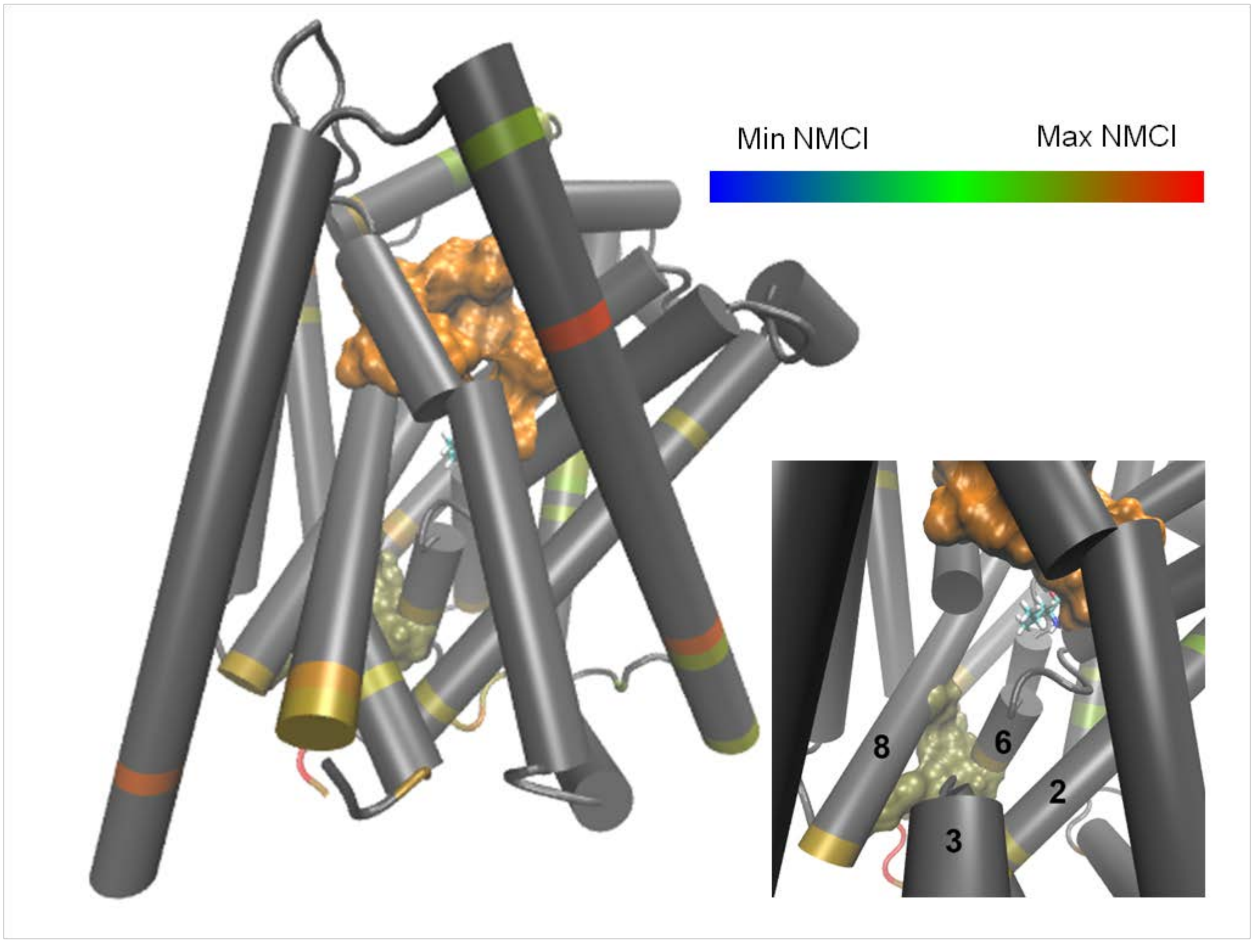

Figure S11. No clear coordination channel is resolvable between S2 and the INI in LeuT$_{\mathbf{MNG\text{-}3}}$. Main: Residues found to have high *mutual coordination information* with S2 and the INI are colored by their *normalized mutual coordination information* (NCMI) using the scale at the top right, where the Min and Max NCMI refer to the minimum and maximum values among all possible residues. All other residues are represented in grey. Bottom right: A close-up of the TM2, TM6b, and TM8 interface.

SUPPORTING REFERENCES

1. Quick M, Winther A-ML, Shi L, Nissen P, Weinstein H, et al. (2009) Binding of an octylglucoside detergent molecule in the second substrate (S2) site of LeuT establishes an inhibitor-bound conformation. Proc Natl Acad Sci U S A 106: 5563–5568. doi:10.1073/pnas.0811322106.

2. Mondal S, Khelashvili G, Shi L, Weinstein H (2013) The cost of living in the membrane: a case study of hydrophobic mismatch for the multi-segment protein LeuT. Chem Phys Lipids 169: 27–38. doi:10.1016/j.chemphyslip.2013.01.006.

3. Martínez L, Andrade R, Birgin EG, Martínez JM (2009) PACKMOL: a package for building initial configurations for molecular dynamics simulations. J Comput Chem 30: 2157–2164. doi:10.1002/jcc.21224.

4. **S**uite 2012: Maestro, version 9.3, Schrödinger, LLC, New York, NY, 2012

5. Yesselman JD, Price DJ, Knight JL, Brooks CL (2012) MATCH: an atom-typing toolset for molecular mechanics force fields. J Comput Chem 33: 189–202. doi:10.1002/jcc.21963.

6. Bai J (2011) Estimating High Dimensional Covariance Matrices and its Applications. Ann Econ Financ 215: 199–215.

7. Gur M, Zomot E, Bahar I (2013) Global motions exhibited by proteins in micro- to milliseconds simulations concur with anisotropic network model predictions. J Chem Phys 139: 121912. doi:10.1063/1.4816375.

8. Kunsch H (1989) The jackknife and the bootstrap for general stationary observations. Ann Stat.

9. Reva B, Antipin Y, Sander C (2007) Determinants of protein function revealed by combinatorial entropy optimization. Genome Biol. doi:10.1186/gb-2007-8-11-r232.

10. Karmeshu, editor (2003) Entropy Measures, Maximum Entropy Principle and Emerging Applications. Vol. 119. Springer.

11. Lange OF, Grubmüller H (2006) Generalized correlation for biomolecular dynamics. Proteins 62: 1053–1061. doi:10.1002/prot.20784.
